# Single‐cell multi‐omics characterize colorectal tumors, adjacent healthy tissue and matched (tumor) organoids identifying CRC‐unique features

**DOI:** 10.1002/ijc.70103

**Published:** 2025-08-23

**Authors:** Zhijun Yu, Merel Derksen, Brigit M. te Pas, Sabrina Ladstätter, Rene Overmeer, Peter Brazda, Marc van de Wetering, Farzin Pourfarzad, Robert G. J. Vries, Wout Megchelenbrink, Christoph Bock, Lucia Altucci, Hendrik G. Stunnenberg

**Affiliations:** ^1^ Princess Maxima Center for Pediatric Oncology Utrecht The Netherlands; ^2^ Department of Precision Medicine University of Campania “Luigi Vanvitelli” Naples Italy; ^3^ Department of Molecular Biology, Faculty of Science Radboud Institute for Molecular Life Sciences, Radboud University Nijmegen The Netherlands; ^4^ HUB Organoids B.V., Utrecht, the Netherlands, part of the Life Science Business of Merck KGaA Darmstadt Germany; ^5^ CeMM Research Center for Molecular Medicine of the Austrian Academy of Sciences Vienna Austria; ^6^ Center for Translational Immunology University Medical Center Utrecht, Utrecht University Utrecht The Netherlands; ^7^ Medical University of Vienna Institute of Artificial Intelligence, Center for Medical Data Science Vienna Austria; ^8^ BIOGEM Ariano Irpino (AV) Italy; ^9^ UP Medical Epigenetics AOU Vanvitelli Naples Italy

**Keywords:** colorectal cancer (CRC), epigenomics, patient‐derived organoids (PDOs), single‐cell analysis, transcriptomics

## Abstract

Colorectal cancer (CRC) arises in the colorectal tissue driven by genetic disorder or the accumulation of somatic mutations, leading to abnormal epithelial cell growth. In this study, we employed single‐nucleus multi‐omics analysis, including single‐nucleus RNA‐seq and single‐nucleus ATAC‐seq, on over 100,000 high‐quality nuclei to investigate the molecular landscape of both primary tissue and patient‐derived organoids (PDOs). Our analysis showed that normal PDOs (N‐PDOs) derived from tissue adjacent to tumors replicate the cellular composition and differentiation trajectory of colorectal crypts. In contrast, tumor PDOs (T‐PDOs) showed patient‐specific transcriptomic and epigenomic heterogeneity yet consistently maintained a stem cell‐like state. T‐PDOs retained the somatic mutation profile of the primary tumor while also exhibiting de novo mutations not detected in either the primary tumor or N‐PDOs. Notably, inferred cell–cell interaction analysis highlighted the activin signaling pathway as a potential unique feature of fibroblast‐epithelial interactions within the tumor microenvironment. This study provides a comprehensive view of the transition from normal to malignant colorectal epithelium and underscores the utility of PDOs as a faithful model for capturing both conserved and patient‐specific features of colorectal cancer.

## What's New?

Patient‐derived organoids provide a promising platform for modeling colorectal cancer. The authors demonstrated that organoids derived from normal colorectal tissue recapitulate the cellular composition and differentiation trajectory of colorectal crypts. Organoids from colorectal tumor tissue exhibited patient‐specific heterogeneity while maintaining a transcriptomic stem‐like state. Notably, the tumor organoids retained the somatic mutation landscape of the primary tumor but also harbored de novo somatic mutations. This study provides a comprehensive view of the transition from normal to malignant colorectal epithelium and underscores the utility of patient‐derived organoids as a faithful model for capturing both conserved and patient‐specific features of colorectal cancer.

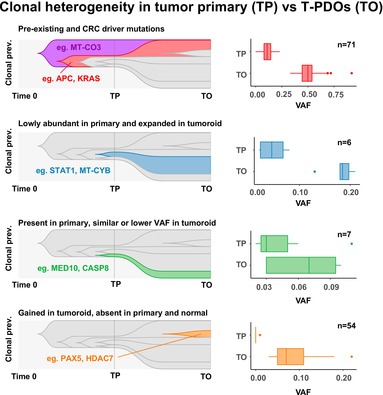

**Abbreviations**
CAF: cancer‐associated fibroblasts
cCDM: combination colon differentiation medium
CNM: colon normal medium
CRC: colorectal cancer
CTM: colon tumor medium
DARs: differential accessible regions
DEGs: differentially expressed genes
ECM: extra cellular matrix
EMT: epithelial–mesenchymal transition
FANS: fluorescence‐activated nucleus sorting
GEM: gel beads in emulsion
GO: Gene Ontology
GSEA: Gene Set Enrichment Analysis
HLA: Human Leukocyte Antigens
iCNV: inferred copy number variation
MMPs: matrix metalloproteinases
MPs: meta‐programs
MSI: microsatellite instability
MSS: microsatellite stable
NES: normalized enrichment scores
NMF: non‐negative matrix factorization
N‐PDO: normal patient‐derived organoids
PCA: principal component analysis
SNP: single nucleotide polymorphisms
TA: transit amplifying
TME: tumor microenvironment
T‐PDO: tumor patient‐derived organoids
TSS: transcription start site
UMAP: Uniform Manifold Approximation and Projection
WNN: weighted nearest neighbor

## 1 INTRODUCTION

Colorectal cancer (CRC) is the third most commonly diagnosed cancer (9.6%) and the second leading cause of cancer‐related mortality (9.3%).^1^ The 5‐year survival rate for CRC is 80%–90% in early stages; that drops to 13% in late stages. Metastasis and treatment resistance are the primary culprits of mortality in late‐stage CRCs, underscoring the need for a deeper understanding of CRC tumor biology.

A key genomic feature in CRC is microsatellite instability (MSI), which arises from the DNA mismatch repair system, involving genes such as *MLH1*, *PMS2*, *MSH6*, or *MSH2*, leading to the accumulation of errors during DNA replication.^2^ MSI is observed in approximately 15% of CRC cases, while the majority (85%) are classified as microsatellite stable (MSS).^3^ Immune checkpoint inhibitor therapy has yielded encouraging outcomes for patients with MSI CRC,^4^ but is largely ineffective in late‐stage MSS CRC due to a lack of robust immune infiltration.^5^ This highlights the importance of characterizing at a finer resolution, particularly in MSS CRCs.

While single‐cell transcriptomic technologies have provided insights into CRC heterogeneity,^6^, ^7^ relatively few studies have explored the epigenome landscape. Furthermore, existing work has tended to focus either on primary tissue^7^ or organoids^8^ in isolation, rather than directly combining and comparing organoids with their matched primary tissues. Given that organoid cultures are often optimized for cell proliferation rather than for promoting cell type maturation through differentiation, it remains unclear how faithful organoids reflect their tissue of origin.

To address this, we employed single‐nucleus multi‐omics, which combines single‐nucleus RNA sequencing (snRNA‐seq) and single‐nucleus assay for transposase‐accessible chromatin using sequencing (snATAC‐seq), on primary normal and tumor tissue and their respective derived N‐PDOs and T‐PDOs. This approach enabled us to assess the extent to which PDOs reproduce the cellular, genetic, transcriptomic, and epigenomic features of primary colorectal tissue, with particular focus on characterizing differentiation trajectories, patient‐specific heterogeneity, and cell–cell interactions within the tumor microenvironment.

## 2 METHODS

This section provides a brief overview of the methods, with detailed descriptions available in Supporting Information.

### 2.1 Primary and PDOs material

This research involves nine patients, aged between 55 and 89, who were diagnosed with colorectal cancer in various hospitals near Utrecht, NL (University Medical Centre Utrecht and Meander Medical Centre [Amersfoort]). Table S1 provides detailed patient metadata, including gender, age, tumor location, pre‐treatment, pathology (WHO) and TNM stage for all nine patients. Following tissue dissociation, normal colon crypts and tumor cell suspensions were partially utilized for organoid establishment. Normal crypts and tumor cells were embedded in Matrigel (Corning) and expanded in colon normal medium (CNM) or colon tumor medium (CTM), respectively (Table S2).

### 2.2 Single‐nucleus RNA and ATAC libraries preparation and sequencing

The buffers used for single‐nucleus suspension preparation are detailed in Tables S3–S7. snRNA and snATAC libraries were prepared according to the protocol provided by the vendor using the 10x Genomics Chromium Next GEM Single Cell Multiome ATAC + Gene Expression kit (CG000338 RevE). The libraries were sequenced on the Illumina NovaSeq 6000 platform at Prinses Maxima Center for Pediatric Oncology (PMC), using a paired‐end and dual indexing setup as recommended by 10x Genomics.

### 2.3 Pre‐processing single‐nucleus RNA and ATAC sequencing data

The Cell Ranger ARC 2.0.2 pipelines (from 10x Genomics) were used to process the multi‐omics sequencing data. The pipelines first demultiplexed the BCL files into FASTQ files, which were then converted into BAM files and feature‐barcode matrices. A multiplexing strategy was used in this project, where samples from two individual patients were pooled prior to library preparation within a 10x Genomics Multiome kit reaction. A single nucleotide polymorphism (SNP)‐based demultiplexing strategy was used to separate two pooled patients from one library.

### 2.4 snRNA‐seq data processing

snRNA‐seq data were processed sequentially from integration, clustering, cell type annotation to downstream cell type proportion analysis, Differentially Expressed Genes (DEGs) analysis, gene ontology (GO) analysis, Gene Set Enrichment Analysis (GSEA), and Non‐negative Matrix Factorization (NMF) analysis, pseudo‐time trajectory analysis, cell–cell interaction analysis, inferred copy number variation analysis, and snRNA‐seq based MSI detection.

### 2.5 snATAC‐seq data processing

snATAC‐seq data were processed from integration, clustering, peak calling, to downstream functional analysis such as Differential Accessible Regions (DARs) analysis, motif analysis, and closest gene analysis.

The sequencing coverage and quality statistics for each sample (if applicable) are summarized in Tables S8–S11.

## 3 RESULTS

**FIGURE 1:**
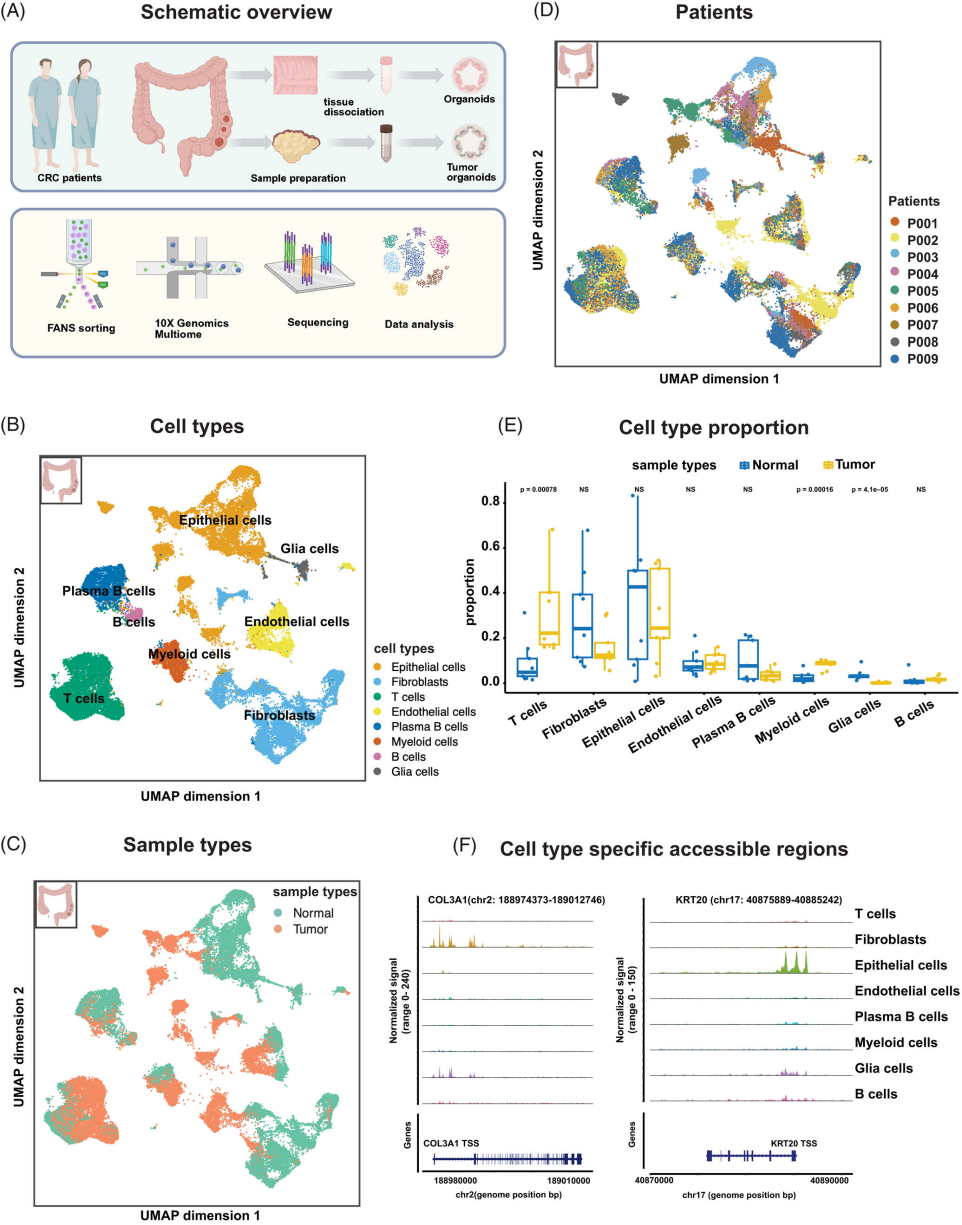
Multi‐omics profiling of primary CRC samples. (A) Schematic overview of the experimental workflow, including tissue dissociation, organoid generation, fluorescence‐activated nucleus sorting (FANS), library preparation, sequencing, and data analysis. (B–D) UMAP embedding of 43,804 nuclei from tumor and adjacent normal tissue after integration of snRNA‐seq and snATAC‐seq data. Cells are colored by cell types (B), sample types (C) and patients (D). The colon icon in the top left corner represents that the data originate from primary colorectal tissue samples. (E) Box plot showing the proportion of each cell type in normal (blue) and tumor (yellow) tissue across patients; each dot represents one patient. Statistical significance was assessed using the Wilcoxon test. NS: non‐significance. (F) Genome coverage plots displaying Tn5 insertion frequency in the genome region of specific genes *COL3A1* and *KRT20*, across different cell types. The transcription start site (TSS) was indicated for those genes. Peak heights represent normalized signals of fragments.

To explore the intricacies of CRCs, we undertook a comprehensive single‐nucleus multi‐omics approach by generating both single‐nucleus transcriptome and single‐nucleus epigenome profiles from the same nucleus. The dataset encompassed four distinct sample types: primary tumor tissue, the adjacent normal tissue (mostly crypts), and their derived N‐PDOs and T‐PDOs (Figure 1A) from nine patients (P001‐P009) with metadata detailed in Table S1. For each sample type, tissue or organoids were dissociated (see Section 2); nuclei were isolated and sorted. snRNA‐seq and snATAC‐seq libraries were generated using the 10X Genomics Multiome platform. Single nucleotide polymorphism (SNP)‐based demultiplexing^9^ was performed on each modality, and the results were concatenated for each library to distinguish patients (see Section 2). Ambient RNA and doublets were filtered before the integration of snRNA‐seq and snATAC‐seq datasets. The weighted nearest neighbor (WNN)^10^ method was used to integrate these two modalities into a unified multimodal analysis.

### 3.1 Integrated multi‐omics reveals cell‐type specific and distinct profiles between normal and CRC primary samples

Focusing our analysis initially on the primary tissue, we generated integrated multi‐omics data of 43,804 high‐quality cells. The cells were then categorized into major cell types based on canonical cell type markers^6^ as depicted in Figure S1A. The number of counts from snRNA‐seq and snATAC‐seq data of each patient after filtering (Figure S1B) resulted in high quality, with an average count above 1000. Dimensionality reduction using Uniform Manifold Approximation and Projection (UMAP) yielded a joint embedding annotated with major cell types (Figure 1B). Overlaying sample type (tumor or adjacent normal sample) onto the UMAP (Figure 1C) revealed limited overlap between normal and tumor samples within epithelial and fibroblast populations, underscoring their distinct transcriptome and epigenome states. Projection of patient distribution (Figure 1D) revealed co‐clustering with respect to T cells and myeloid cells, whereas epithelial cells and fibroblast populations exhibited patient‐specific clustering patterns, indicating inter‐patient heterogeneity in those compartments. A comparative analysis of the cell type distribution between adjacent normal and tumor samples revealed significant alterations in cell ratios across cell types (Figure 1E). Notably, T cells, myeloid cells, and glial cells exhibited considerable variation in their proportions. The cell type distribution highlighted patient‐specific differences in the proportion of a given cell type (Figure S1C).

To investigate the variations among cell clusters in terms of chromatin accessibility, differentially accessible regions (DARs) were determined per cell type using the FindAllMarkers function from the R package Signac.^11^ Log‐fold change (avg_log2FC >1) and adjusted *p*‐value (*p*_val_adj <.001) were used to select the significant DARs. Figure S1D shows the selected most significant DARs for each cell type and lists the top 5 closest genes. The DARs relate to differentially expressed genes; for example, *COL3A1* and *KRT20* were highly accessible for fibroblast and epithelial cells, respectively (Figure 1F), in line with their high expression levels (Figure S1A). In summary, we observed cell‐type specific differences in the transcriptome (Figure S1A) and epigenome (Figures S1D and 1F), and distinct profiles between colorectal normal and tumor tissue (Figure 1C,D).

### 3.2 Identification of CRC‐specific clusters with chromosomal‐CNV, regulatory motifs in tumor progression, stemness, and BEST4 cell differentiation

**FIGURE 2:**
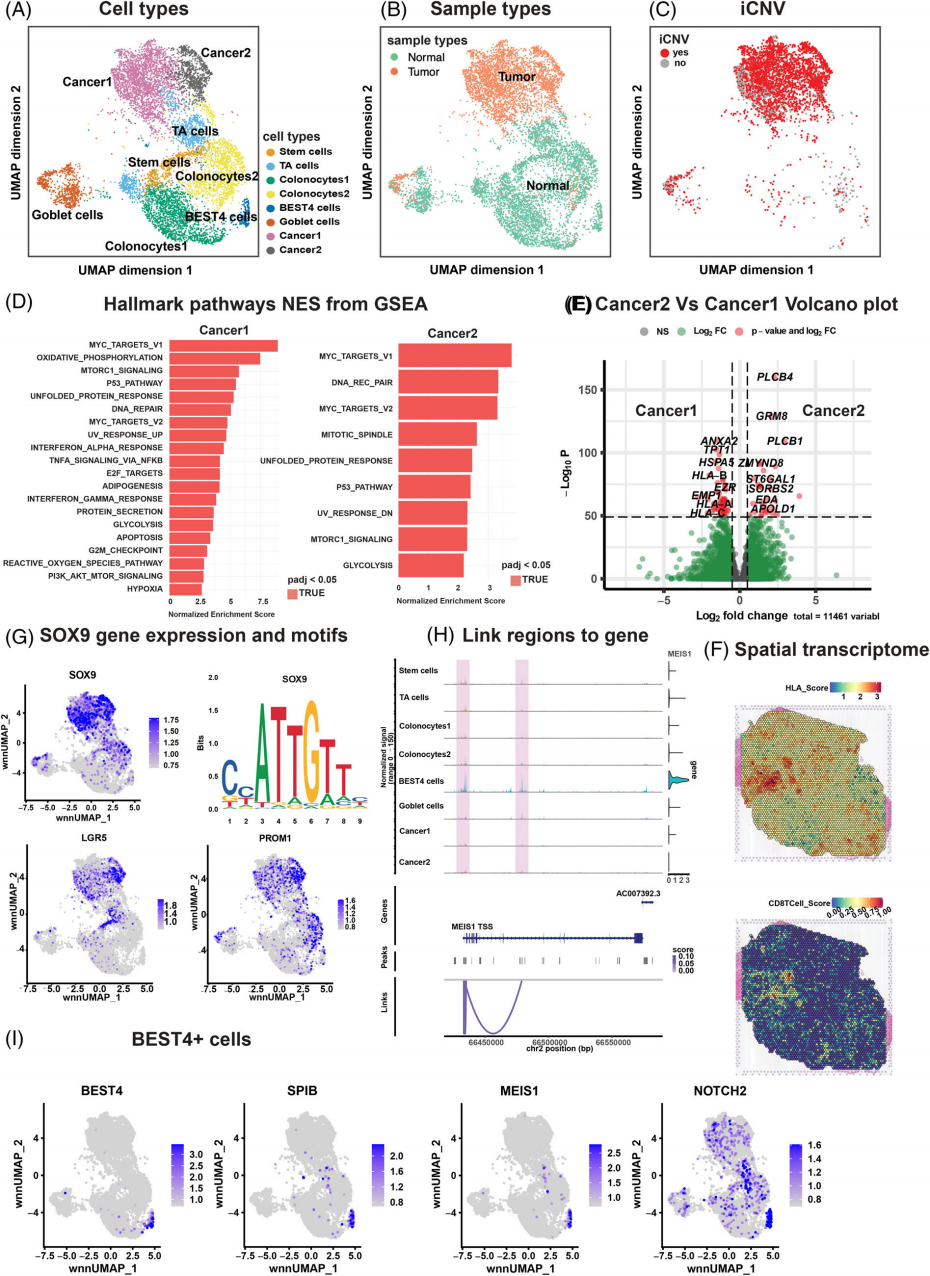
Tumor cell characteristics in CRC. (A, B) UMAP projection of 8245 nuclei from the epithelial compartment (including both tumor and adjacent normal tissues), after integration of batch‐corrected snRNA‐seq and snATAC‐seq data. Cells are colored by cell types (A) and sample types (B). (C) UMAP of 3624 epithelial nuclei from tumor tissue only, colored by inferred copy number variation (iCNV) status: Red indicates the presence of iCNVs and gray indicates none. (D) Gene Set Enrichment Analysis (GSEA) for hallmark pathways in Cancer1 and Cancer2 clusters. Red bars indicate significantly enriched pathways (adjusted *p*‐value, *p*‐adj <.05). (E) Volcano plot displaying the differentially expressed genes between the Cancer2 and Cancer1 clusters. Red dots indicate genes with an adjusted *p*‐value of log fold change greater than 50 times, and green dots indicate genes less than 50 times. (F) Spatial transcriptome showing the HLA class I and CD8+ T cell score, highlighting regions with elevated expression of HLA I and CD8+ T cell markers. (G) UMAP plots showing normalized gene expression levels of *SOX9*, *LGR5*, and *PROM1*. The top‐right plot displays the position weight matrices for the *SOX9* motif. (H) Chromatin accessibility peaks associated with *MEIS1* expression across ±20 kb of gene body. High‐confidence regulatory regions (adjusted for bias with region size, GC content and overall accessibility) are highlighted in pink. The transcription start site (TSS) is indicated. (I) UMAP plots showing expression of *BEST4*, *SPIB*, *MEIS1*, and *NOTCH2*. Color scale represents the normalized log‐fold change.

The distinct transcriptional and epigenetic profiles observed between normal and tumor epithelial cells (Figure 1B,C) prompted us to focus on the epithelial compartment, which included 8245 nuclei for further analysis. Batch correction^12^ was applied separately on snRNA‐seq and snATAC‐seq datasets, followed by integrating using the WNN method.^10^ The resulting UMAP (Figure 2A) revealed an epithelial landscape, including stem cells, transit amplifying (TA) cells, colonocyte subtypes (colonocytes1 and colonocytes2), BEST4 cells (Bestrophin 4 highly expressed), goblet cells, and two tumor‐specific clusters, designated Cancer1 and Cancer2. The marker genes expression profiles for these populations are illustrated as a dot plot (Figure S2A). Cells from normal and tumor tissues displayed distinct distributions across clusters (Figure 2B). Cancer1 and Cancer2 clusters predominantly consist of cells derived from tumor samples, as revealed by the examination of cell type distribution between normal and CRC tissues (Figure S2B) and the ratios of cell types per patient (Figure S2C). This finding piqued our interest in investigating the chromosomal copy number states of these cells. Therefore, matched snRNA‐seq data was used to get the inferred copy number variation (iCNVs) via the R package Numbat.^13^ Figure 2C presents the iCNV states of the tumor samples on a UMAP, illustrating that most cells in the Cancer1 and Cancer2 clusters possess at least one iCNV (red dots), confirming their tumor cell identity. The iCNV burden varied across patients (Figure S2D). For instance, P008 and P009 exhibit alterations across at least 15 chromosomes, whereas only a few chromosomes with iCNVs were detected in patients P006 and P007. Additionally, amplification of chromosome 20q and chromosome arm 8q amplification were present in seven and five out of nine patients, respectively. Chromosome 8q amplification is commonly observed in CRCs, whereas chromosome 20q amplification is more frequent in MSS CRCs.^14^ MSI/MSS status, determined via MSIsensor‐RNA package,^15^ classified all 9 patients as MSS (Table S12).

To distinguish functional differences between the Cancer1 and Cancer2 clusters, we performed gene set enrichment analysis (GSEA)^16^ on the snRNA‐seq data to examine the normalized enrichment scores (NES) for hallmark pathways. Notably, several hallmark pathways were significantly enriched (*p*‐adj <.05, NES >2), including *P53*, oxidative phosphorylation, *mTOR*, and *MYC* target gene sets (Figure 2D). *MYC* was highly expressed in both cancer clusters (Figure S2E), and *MYC* is known to play a crucial role in cellular processes such as cell cycle progression, apoptosis, and metabolism, making it a key factor in cancer development and progression.^17^ Likely, *MYC* also plays pivotal roles in CRC development. A volcano plot analysis shows the differences between Cancer1 and Cancer2 clusters (Figure 2E). The Cancer2 cluster showed an enrichment for phosphoinositide cycle‐related genes *PLCB4* and *PLCB1*, suggesting an enhanced migration potential of these cells and a poor overall survival rate.^18^ The Cancer1 cluster is enriched with Human Leukocyte Antigens (HLA) class I genes, including *HLA‐A*, *HLA‐B*, and *HLA‐C*, which may imply the potential for recognition by CD8+ T cells.^19^ To explore this further, spatial transcriptome data^20^ were analyzed for co‐localization between HLA expression and CD8+ T cell scores (see Section 2). The results (Figure 2F) revealed a correlation coefficient of 0.3, supporting potential spatial proximity between tumor cells and T cells. Additionally, the expression levels of marker genes such as *EPCAM*, *SOX9*, and *CD8A* are shown in Figure S2F.

Chromosome accessibility analysis within the epithelial dataset identified differentially accessible regions (DARs) per cell type, with corresponding enriched motifs and nearby gene annotations (Figure S2G). The analysis revealed, among others, *SMOC2* for stem cells, *BEST4* for BEST4 cells, and *ATOH1* for goblet cells, in line with their expression levels in corresponding clusters (Figure S2A). Intriguingly, motifs of the *FOS*/*JUN* gene family were top‐ranking in the Cancer1 cluster, suggesting cellular stress responses,^21^ while Cancer2 was enriched for *TCF* and *SOX* family motifs (Figure 2G, upper panel), particularly SOX9, which was highly expressed in both cancer clusters. *SOX9* is recognized as a cancer stem cell marker, promoting CRC by blocking differentiation via up‐regulating *PROM1* to support stem cell signaling.^22^ *PROM1* exhibited high gene expression levels (Figure 2G, lower panel) in Cancer1 and Cancer2 clusters, supporting the existence of a potential *SOX9*‐*PROM1* regulatory axis. The stem cell marker *LGR5* showed elevated expression in Cancer2 when compared to the Cancer1 cluster, implying pro stem‐state features of the Cancer2 cluster.

We also investigated the regulatory landscape of BEST4 cells, a recently identified^6^ mature epithelial subtype in the human intestine. Tania and colleagues^23^ provided a comprehensive review of BEST4 cells in the intestinal epithelium, highlighting varying abundancies across gut segments, and suggested a role in pH regulation, electrolyte transport, and epithelial defenses. Motif analysis and gene expression pointed to the transcription factors, *SPIB* and *MEIS1*, as potential transcriptional regulators (Figure S2A). The *SPIB* gene is a known marker for BEST4 cells and is reported to play important roles in cell type development.^23^ *MEIS1* is reported to be an oncogene in leukemia.^24^ We observed that chromatin accessibility near the transcription start site (TSS) and within the intronic region of *MEIS1* (Figure 2H) and *SPIB* (Figure S2H) was strongly correlated with their expression levels, suggesting that promoter and/or enhancer activity is involved in modulating BEST4. High *NOTCH2* expression (Figure 2I) further supports BEST4 cell absorptive lineage potential, consistent with the role of NOTCH signaling^25^ in driving absorptive differentiation.

Collectively, this analysis reveals distinct transcriptional and epigenetic profiles between normal and tumor epithelial cells in CRC, identifies cancer‐specific clusters with chromosomal copy number variations, and highlights the potential regulatory genes and motifs involved in tumor progression, stemness, and BEST4 cell differentiation.

### 3.3 Multi‐omics approach suggests versatile roles of CAFs in CRC

**FIGURE 3:**
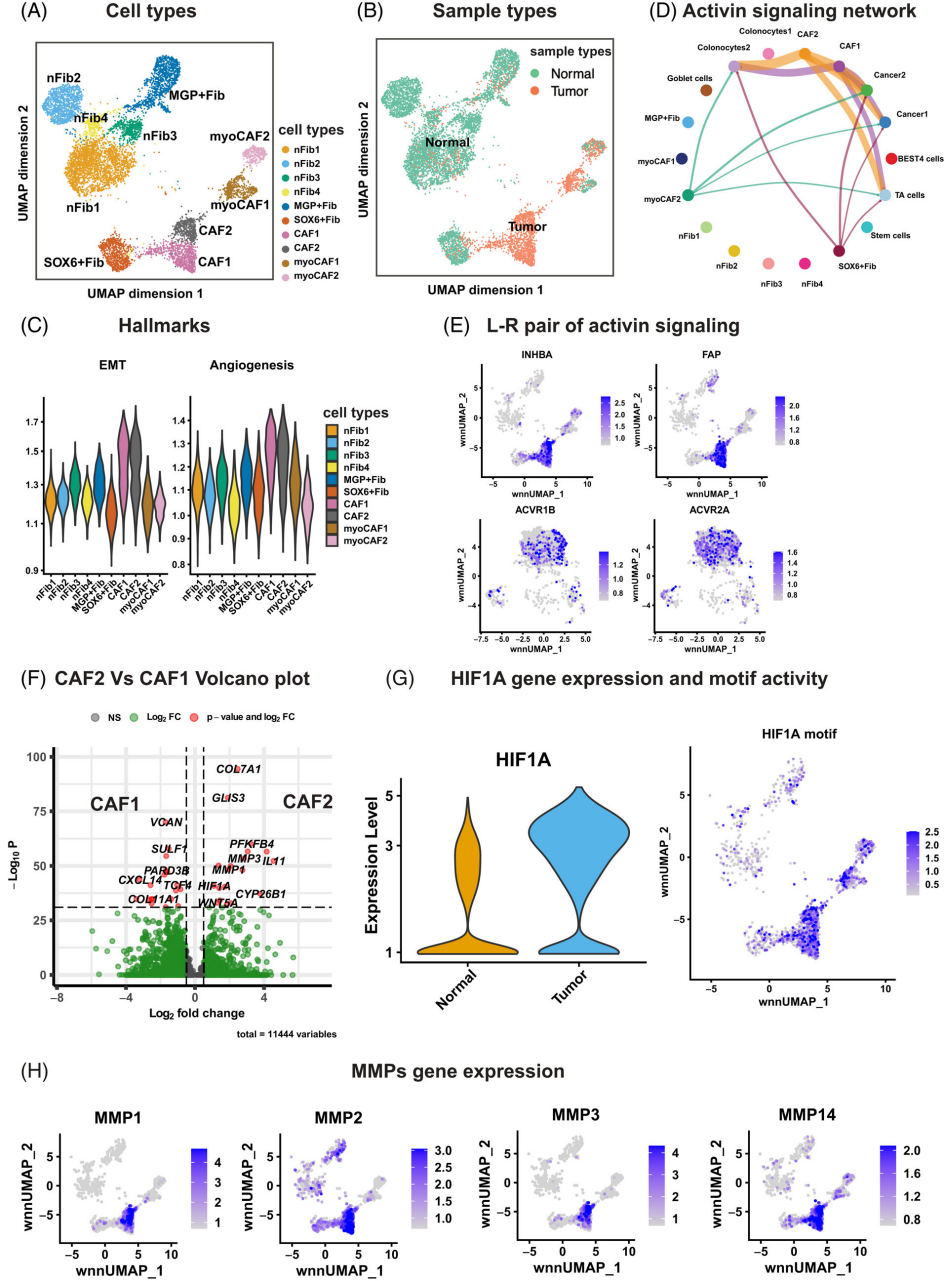
Characterization of cancer‐associated fibroblasts (CAFs) in CRC. (A, B) UMAP projection of 8151 nuclei from the fibroblast compartment (including both tumor and adjacent normal tissue) after integration of batch‐corrected snRNA‐seq and snATAC‐seq data. Cells are colored by cell types (A) and sample types (B). “n,” represents normal; “Fib” represents fibroblast; “CAF” represents cancer‐associated fibroblasts; and “myo” represents myofibroblast‐like. (C) Violin plots displaying Gene Set Enrichment Analysis (GSEA) scores for epithelial–mesenchymal transition (EMT) and angiogenesis hallmark pathways across fibroblast subclusters. (D) Circle plot illustrating inferred activin signaling interactions between epithelial and fibroblast subclusters in tumor samples. Nodes represent cell types; edge widths reflect the number of predicted ligand‐receptor interactions, and edge color corresponds to the ligand‐producing cell type. (E) UMAP plots displaying normalized gene expression levels of *INHIBA* and *FAP* in the fibroblasts (top panel) and *ACVR1B* and *ACVR2A* in epithelial cells (bottom panel) from tumor samples. (F) Volcano plot showing the differentially expressed genes between the CAF2 and CAF1 clusters. Red dots indicate genes with an adjusted *p*‐value of log fold change greater than 30 times, and green dots indicate genes less than 30 times. (G) Left: violin plot comparing *HIF1A* expression in fibroblasts from normal and tumor tissues. Right: UMAP plot displaying *HIF1A* motif enrichment in fibroblasts from tumor samples. (H) UMAP plots displaying normalized gene expression levels of *MMP1*, *MMP2*, *MMP3*, and *MMP14* across fibroblasts in tumor samples.

Fibroblasts were found to exhibit distinct transcriptional and epigenomic profiles between normal and tumor tissues, capturing our interest (Figure 1B,C). We subselected the fibroblasts for in‐depth analysis. To this end, snRNA‐seq and snATAC‐seq datasets were independently batch‐corrected and subsequently integrated (WNN method^10^), followed by UMAP visualization with annotated cell types (Figure 3A). Consistent with published data,^26^ we observed high expression of the fibroblast activation protein (*FAP*), actin alpha 2 (*ACTA2*) and platelet‐derived growth factor subunit A (*PDGFA*) in fibroblasts derived from tumor samples (Figures 3B and S3A), which we refer to as Cancer‐Associated Fibroblasts (CAFs). Based on the clustering, differentially expressed genes (DEGs) and sample types, we grouped fibroblasts into several clusters: normal fibroblasts1‐4 (nFib1‐4), *MGP* or *SOX6* gene highly expressed fibroblasts (MGP+ Fib and SOX6+ Fib), CAF1, CAF2, myofibroblast‐like CAFs (myoCAF1‐2) (Figures 3A and S3B). A comparative analysis of cell type distribution between normal and tumor fibroblast tissue (Figure S3C) revealed that the CAF1 and CAF2 were predominately composed of cells derived from tumor samples. Given the crucial role of CAFs in promoting tumor growth,^27^ facilitating angiogenesis,^28^ and aiding invasion and metastasis,^29^ we focused our analysis on these clusters.

Hallmark pathways analysis using the Molecular Signatures Database (MSigDB)^16^, ^30^ showed that epithelial–mesenchymal transition (EMT) and angiogenesis programs were elevated in CAF1 and CAF2 compared to the other fibroblast subclusters (Figure 3C). To further explore the potential roles of CAFs in modulating EMT and angiogenesis in colorectal cancer, we applied CellChat^31^ to infer ligand‐receptor interactions in normal and tumor samples, respectively. Notably, the activin (*INHBA*‐[*ACVR1B* + *ACVR2A*]) signaling pathway appeared to be nearly exclusively expressed in tumor samples, suggesting a possible communication route between fibroblasts and epithelial cells (Figure S3D).

Focusing on fibroblast‐epithelial interactions, we applied our cell–cell interaction analysis exclusively on the fibroblast and epithelial compartments. Interestingly, we found that the activin signaling pathway likely connects CAF1/2 with epithelial tumor clusters (Cancer1/2) (Figures 3D and S3E). The expression of *INHBA*, *ACVR1B*, and *ACVR2A* likely supports a potential tumor‐promoting signaling axis (Figures 3E and S3F).

Comparative gene expression analysis between CAF1 and CAF2 (Figure 3F) revealed distinct molecular programs. CAF2 exhibited higher expression of *HIF1A*, *IL11*, *WNT5A*, *MMP1*, and *MMP3*, while CAF1 was enriched for *CXCL14* and *COL11A1*. *HIF1A*, a known regulator of hypoxia, proliferation, and chemoresistance in CRC,^32^ was significantly elevated in CAFs compared to normal fibroblasts (Figure 3G, left panel) and its motif was enriched in tumor fibroblasts (Figure 3G, right panel). Previous research in breast^33^ and gastric^34^ cancer implicates *HIF1A* in the regulation of matrix metalloproteinases (MMPs), consistent with our findings of high *MMP1*, *MMP2*, *MMP3*, and *MMP14* expression in CAFs (Figure 3H). These MMPs are known to maintain extracellular homeostasis^35^ and may enable fibroblast‐driven cancer cell invasion and metastasis. Moreover, the elevated expression of inflammation mediator interleukin‐11 (*IL11*) and interleukin‐24 (*IL24*) suggests a pro‐inflammatory role of CAF2 (Figure S3G). Importantly, *IL11* has been linked to poor prognosis in CRC.^36^ Taken together, CAF2 may encompass inflammation, ECM remodeling, and hypoxia‐associated features, aligning with inflammatory (iCAF), matrix (mCAF) and metabolic (meCAF) subtypes.^37^

In summary, while experimental validation is required, our integrated multi‐omics analysis provides insight into the versatile roles of CAFs in CRC malignancy and highlights *INHBA* and *HIF1A* as candidate therapeutic targets for future investigation.

### 3.4 Comprehensive characterization of cell types and pseudo‐time trajectory of colorectal N‐PDOs

**FIGURE 4:**
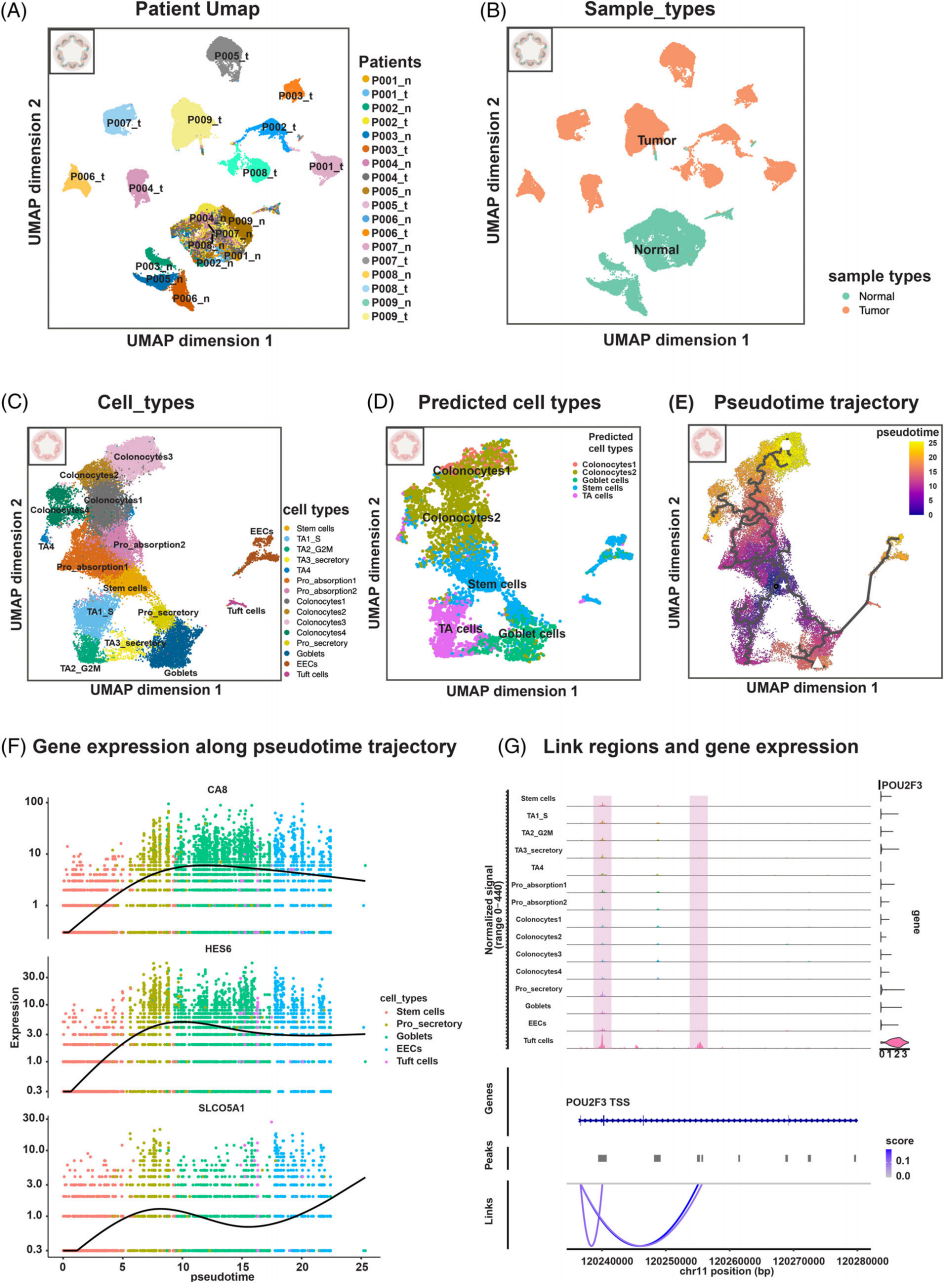
Colorectal N‐PDOs. (A, B) UMAP projection of 65,463 nuclei from organoid samples derived from both tumor and adjacent normal tissue, after integration of snRNA‐seq and snATAC‐seq data. Cells are colored by patients (A) and sample types (B). “n” represents N‐PDOs, “t” represents T‐PDOs. The mixed color organoid icon in the top left corner represents that the data originate from colorectal N‐ and T‐PDOs. (C) UMAP projection of 28,604 nuclei from N‐PDOs based on batch‐corrected snRNA‐seq data, colored by annotated cell types. Abbreviations include: TA1_S (transit amplifying cells in S phase), TA2_G2M (transit amplifying cells in G2M phase), TA3_secretory (secretory lineage related transit amplifying cells), Pro (progenitor state), EECs (enteroendocrine cells). The pink color organoid icon in the top left corner represents that the data originate from colorectal N‐PDOs. (D) UMAP of 5000 nuclei randomly down sampled from the colorectal N‐PDOs snRNA‐seq data, colored by predicted cell types. TA denotes transit amplifying. (E) Pseudo‐time trajectory analysis of N‐PDOs based on snRNA‐seq data. Cells are colored by pseudo‐time score, representing progression from stem‐like to mature cell states. Black lines indicate the structured trajectory branches. The star marks stem cells, the hexagon marks mature absorptive cells, and the triangle marks goblet cells. (F) Gene expression dynamics of *CA8*, *HES6*, *SLCO5A1* across the pseudo‐time trajectory for secretory lineage clusters (including stem cells, pro_secretory, goblets, EECs and tuft cells). Cells are colored by cell types; black lines represent average gene expression trends. (G) Genome coverage plot displaying peaks associated with *POU2F3* gene expression across ±20 kb of gene body. High‐confidence regulatory regions (adjusted for bias with region size, GC content and overall accessibility) are highlighted in pink. The transcription start site (TSS) is indicated.

PDOs derived from CRC tumors (T‐PDOs) and from adjacent normal tissues (N‐PDOs) were initially cultured in expansion medium, followed by a brief 4‐day period in differentiation medium (see Section 2). A schematic overview of the PDO generation workflow is shown in Figure S4A. Both N‐ and T‐PDOs were dissociated and processed to generate single cell multiome libraries. The integrated analysis of snRNA‐seq and snATAC‐seq data across patients and sample origins is shown in Figure 4A,B. These visualizations revealed distinct cellular states between N‐ and T‐PDOs, as well as heterogeneity among T‐PDOs. Interestingly, six N‐PDOs derived from left‐sided colorectal tissue (P001‐P002, P004 and P007‐P009) appeared to cluster together in the UMAP space, whereas three PDOs from the right‐sided tissue (P003, P005‐P006) formed a separate group (Figure 4A and Table S1), potentially reflecting regional differences in cell states. In contrast, T‐PDOs tend to form distinct clusters per patient, suggesting variability in transcriptional and epigenomic profiles among patients (Figure 4A).

To explore the cellular composition of N‐PDOs, we performed clustering and cell type annotation using only the N‐PDOs subset. This analysis revealed multiple cell types typically associated with colorectal normal epithelium^6^ (Figure 4C); however, we did not observe BEST4 cells or rare M(microfold)‐like cells. A comparison of cell‐type proportions between right‐ and left‐sided N‐PDOs (Figure S4B) suggested that right‐sided N‐PDOs are enriched in mature absorptive cells (colonocytes1,2,3), whereas left‐sided N‐PDOs appeared to contain more secretory lineage cells (pro‐secretory, goblets and tuft cells), implying a possible difference in lineage differentiation bias between regions.

We next compared colorectal N‐PDOs with matched normal primary tissue using snRNA‐seq data. Using anchor‐based reference mapping*(10)*, we predicted cell types in N‐PDOs based on a reference from normal epithelial cells. Predicted annotations (Figure 4D) generally agreed with manual curation, except for EECs and tuft cells that were misassigned to stem cells, likely due to low expression of key stem markers (*LGR5* and *SMOC2*) in these populations (Figure S4C).

We next assessed the potential developmental trajectory in N‐PDOs from stem cell to mature states. Expression of lineage markers *HES6* ^38^ (secretory lineage marker) and *UGT1A8* (absorptive lineage marker) was assessed to distinguish lineage branches (Figure S4D). Using Monocle3,^39^ we inferred a pseudotime trajectory resembling crypt development,^40^ which suggested progression from the stem cells (star) to mature absorption lineage colonocytes (hexagon), with a branch path toward mature secretory lineage cells including goblets (triangle), EECs, and tuft cells (Figure 4E). Gene expression patterns of secretory‐associated genes (*CA8*, *HES6* and *SLCO5A1*) and absorptive markers (*FABP1*, *PAG1* and *SI*) along the pseudotime trajectory appear consistent with these inferred lineages (Figures 4F and S4E).

On the chromatin level, we determined DARs, the closest features, and enriched motifs by cell type (Figure S4F). *TCF*‐family motifs were among the most enriched in stem cells and TA2_G2M clusters, aligning with their known role in proliferation via the canonical Wnt/beta‐catenin pathway.^41^ Transcription factor DNA binding motifs for *NEUROD1* and *NEUROG2* were enriched in secretory lineages (e.g., TA3_secretory, Pro_secretory and EECs), aligning with their known involvement in endocrine specification.^42^, ^43^ The transcriptomics data confirm the expression of *NEUROD1* in EECs (Figure S4G). The motifs of transcription factors from the POU family were prominent in tuft cells, with *POU2F3* appearing as both enriched motif and closest feature (Figure S4E). Tuft cells are chemosensory cells that play roles in responding to foreign pathogens, and *POU2F3* is reported as a master regulator in differentiation into tuft cells.^44^ Linking gene expression with chromatin accessibility (gene body ±20 kb) revealed regions (pink rectangles) with high accessibility in tuft cells that were strongly associated with *POU2F3* expression (Figure 4G), suggesting these may function as candidate regulatory elements.

In summary, our single‐nucleus multiomic analysis suggests that a range of epithelial cell types are present in N‐PDOs, with transcriptome profiles resembling those of primary normal epithelial cells (Figure 4C,D). The data further imply a possible development trajectory along absorptive and secretory lineages and reveal chromatin features that may be involved in regulating lineage specification. While these findings are observational and require experimental validation, they offer insights into the cellular and regulatory landscape of colorectal PDOs.

### 3.5 Multi‐modal characterization of T‐PDOs reveals transcriptome heterogeneity

**FIGURE 5:**
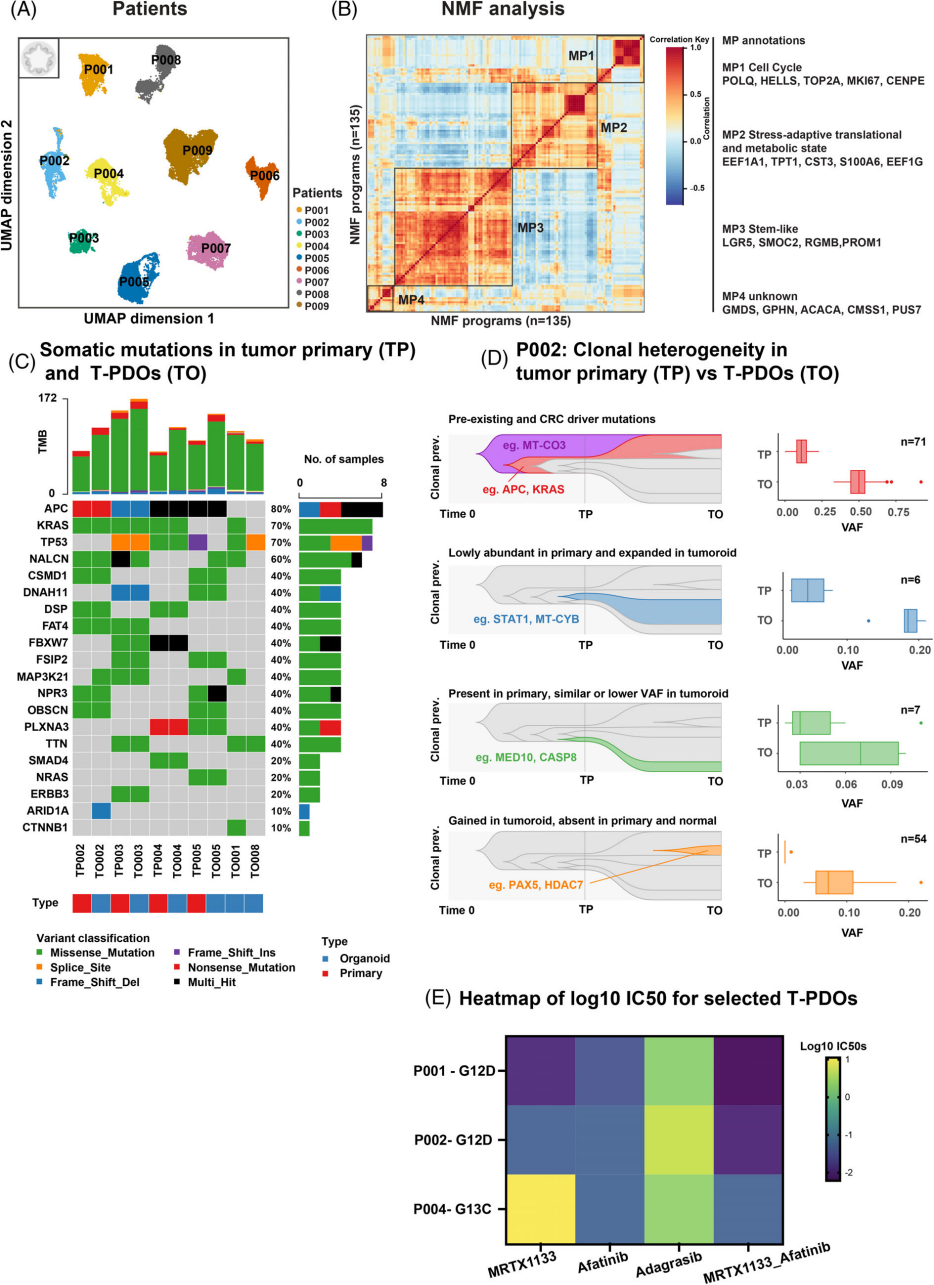
Heterogeneity of colorectal T‐PDOs. (A) UMAP projection of 41,914 nuclei from T‐PDOs, following integration of snRNA‐seq and snATAC‐seq data. Cells are colored by patients. The gray color organoid icon in the top left corner indicates that these data originate from T‐PDOs. (B) Heatmap depicts pairwise Pearson correlation among 135 NMF programs, across patients. Color intensity reflects correlation ranging from −1 to 1. Four distinct meta‐programs (MPs) were identified and are outlined in numbered boxes. Annotations and representative genes for each MP are shown on the right. (C) Oncoplot from WXS showing 20 selected somatic mutations across 6 patients, including paired samples (TP: tumor primary sample and TO: T‐PDOs) and solo samples (only TO available). Mutations with at least 5 reads supporting the ALT allele and VAF ≥0.05 are depicted as squares. Vertical bars depict the number of mutations detected per sample (TMB: tumor mutation burden); horizontal bars depict the relative frequency of a particular mutation across samples. (D) Left: fish plots of WXS inferred clonal evolution in patient P002 from primary tumor (TP) to T‐PDOs (TO). Four (sub)clones in the tumor primary (TP) and T‐PDOs (TO). Right: boxplots of the VAF per mutation in each of these (sub)clones in the tumor primary (TP) and T‐PDOs (TO). The y‐axis depicts the relative clonal prevalence, that is, the relative abundance of cells in the inferred (sub)clone. The red and blue clones were significantly expanded in the T‐PDOs, the green clone remained rare in the T‐PDOs, and the orange clone contains somatic mutations that were unseen in the primary tumor tissue. (E) Heatmap showing the log_10_ IC50 values for selected T‐PDOs with *KRAS* mutations treated with targeted therapies and combinations. Rows represent individual T‐PDOs from P001 and P002 (harboring *KRAS‐G12D*) and P004 (*KRAS‐G13C*). Columns indicate drug treatments: MRTX1133 (a selective *KRAS‐G12D* inhibitor), Afatinib (*EGFR* inhibitor), Adagrasib (*KRAS‐G12C* inhibitor), and respective drug combinations. Color intensity reflects drug sensitivity, with lower values (purple) indicating higher sensitivity, and higher values (yellow) indicating lower sensitivity or resistance.

T‐PDOs displayed higher heterogeneity compared to N‐PDOs (Figure 4A). To gain a deeper insight into this variability, we performed UMAP based on integrated snRNA‐seq and snATAC‐seq data grouped by patient (Figure 5A). DEG analysis highlighted intra‐tumor transcriptional heterogeneity (Figure S5A). Representative DEGs from each T‐PDO were visualized with the four most discriminative genes per sample displayed (Figure S5B).

To uncover the transcriptional features that were shared among T‐PDOs, we applied the Non‐negative Matrix Factorization (NMF)^45^ to the snRNA‐seq data of each T‐PDO using k = 15 factors per sample. This yielded 135 NMF programs across 9 T‐PDOs. The top 50 genes from each NMF program were used to calculate enrichment scores per cell via AddModuleScore.^46^ Pearson correlation analysis of these enrichment scores (Figure 5B, left panel) revealed four distinct meta‐programs (MPs) defined using a hierarchical clustering cutoff height of 1.3. We then selected the top 50 genes (Table S13) most frequently occurring per MP and applied gene ontology analysis to define a biological function associated with each meta program (Figure S5C). MP1, cell cycle: a program enriched for cell proliferation and mitosis‐associated genes (e.g., *MKI67*, *TOP2A*, *CDK1*), making tumor cells actively cycling. The presence of MP1 in some samples could correlate with aggressive growth behavior, suggesting it may serve as a feature of tumor cell proliferation capacity (Figure 5B, right panel). MP2, stress‐adaptive translational and metabolic state: characterized by the enrichment of genes associated with translational control (e.g., *EEF1A1*, *EEF2*, *RACK1*), mitochondrial and metabolic function (e.g., *COX6A1*, *HMGCS1*, *FDPS*) and cellular stress response pathways (e.g., *FTH1*, *MIF*, *GSTP1*). This program likely reflects a cellular state marked by enhanced protein synthesis and metabolic rewiring under stress conditions, potentially supporting survival and plasticity in a hostile tumor microenvironment. MP3, stem‐like program: enriched for genes associated with stem cell maintenance, WNT signaling, and epithelial regeneration consistent with a tumor‐initiating or progenitor‐like state. Clinically, such stem‐like programs are often associated with resistance to therapy and increased metastatic potential, making MP3 of interest for identifying aggressive subpopulations within PDOs. MP4, unknown program: this program lacked consistent enrichment for any known biological process across T‐PDOs, suggesting it may represent either sample‐specific technical variation, an uncharacterized cellular state, or a rare transcriptional program. Together, these MPs provide insights into the functional heterogeneity of T‐PDOs.Projection of these MPs onto the UMAP revealed that MP1 and MP3 dominated most PDOs (P001‐P006, P008‐P009). In contrast, the T‐PDOs of patient P007 were enriched for MP2 and lacked MP3, consistent with a loss of stem‐like features (Figure S5D). Cell type annotations were inferred using an anchor‐based approach^10^ with matched N‐PDOs per patient as reference. The predicted cell type distributions (Figure S6A,B) aligned with NMF findings, with P007 notably lacking stem‐like populations.

In summary, our analysis revealed distinct transcriptional programs across T‐PDOs, highlighting functional heterogeneity and patient‐specific variation. The identification of MPs related to proliferation, stress response, and stemness underscores the potential of T‐PDOs to capture clinically relevant tumor states.

### 3.6 Clonal dynamics and drug sensitivities of T‐PDOs

At the genomic level, we addressed the heterogeneity in primary CRC and T‐PDOs by whole exome sequencing (WXS) using adjacent normal tissue and N‐PDOs as germline controls (see Supporting Information Methods). Our analysis revealed a heterogeneous set of driver and secondary mutations including *APC*, *KRAS*, *TP53*, *CSMD1*, *DSP*, *SMAD4* that were unveiled in both primary tumor and corresponding T‐PDOs (Figure 5C). Variant allele frequencies (VAFs) were generally higher in T‐PDOs compared to primary tissue, consistent with the enrichment of epithelial cells and loss of immune/stromal components in T‐PDOs. Furthermore, significant deviation of primary and T‐PDO VAFs revealed the expansion and loss of specific tumor (sub)clones (Figure 5D). Additionally, we detected “de novo” somatic mutations (e.g., *PAX5* and *HDAC7*) that were exclusive to the T‐PDOs, suggesting ongoing tumor evolution and/or genomic instability.

To improve mutation mapping, we performed full‐length cDNA sequencing using the Oxford Nanopore Technology PromethION (ONT). While the coverage did not allow for confident single‐cell resolution of mutations such as *KRAS* and *TP53* (Figure S7A, left panel), we leveraged a highly expressed mitochondrial mutation naming mitochondrial cytochrome C (*MT‐CO3*) (Figure S7A, right panel). This variant was detected in over 88,000 reads and was present in most tumor organoid cells of patient P002 (Figure S7B–D), implying clonal expansion.

Furthermore, WXS and ONT analysis revealed *KRAS* mutations in T‐PDOs P001, P002 (*KRAS*‐G12D) and in P004 (*KRAS*‐G13C) (Figure S7A,E,F). These three T‐PDO lines were selected for drug screening based on the presence of oncogenic *KRAS* mutations, which are well‐established drivers of tumorigenesis and have been a focus of targeted therapy development. Notably, T‐PDOs P001 and P002, but not P004, were sensitive to the selective *KRAS‐G12D* inhibitor MRTX1133 (Figure 5E), validating the specific action of MRTX1133. Interestingly, all three T‐PDO lines responded to the pan‐ErbB inhibitor Afatinib, even though KRAS mutations are typically associated with resistance to anti‐*EGFR* monoclonal antibodies (like cetuximab and panitumumab).^47^ This sensitivity may be attributed to Afatinib's broader inhibition of the ErbB receptor family, which may suppress compensatory signaling pathways that *KRAS*‐mutant cells rely on to survive. In contrast, none of these T‐PDO lines responded to Adagrasib, a selective *KRAS*‐G12C inhibitor, consistent with the absence of this specific mutation in all three T‐PDOs. These results not only highlight the specificity of *KRAS*‐targeted therapies but also underscore the importance of matching molecular alterations with drug mechanisms. Relative viability of per T‐PDO responds to those drugs and drug combinations is shown in Figure S7G. Overall, the results demonstrate that the T‐PDOs recapitulate known genotype‐specific drug sensitivities, supporting their utility as patient‐specific preclinical models.

In conclusion, integrated genomic and drug response profiling of T‐PDOs revealed extensive intra‐ and inter‐tumoroid heterogeneity, clonal evolution, and mutation‐specific therapeutic sensitivities, highlighting the value of PDOs as a platform for personalized cancer research and precision oncology.

## 4 DISCUSSION

The development of CRC is a complex, multi‐step process that encompasses the accumulation of genetic and epigenetic alterations, along with dynamic cross talks between tumor cells and tumor microenvironment (TME) as well as environmental factors. Understanding this complexity is critical for developing effective, individualized treatment strategies. PDOs provide a promising platform for modeling CRC; whether they fully capture tumor heterogeneity and maintain disease‐relevant features remains to be thoroughly investigated.

In this study, we employed single‐nucleus multi‐omics profiling, combining snRNA‐seq and snATAC‐seq to map the molecular landscapes of primary CRC tissues and matched PDOs. Colorectal N‐PDOs, derived from adjacent non‐tumor tissues, recapitulated the cellular hierarchy and differentiation trajectory of the colorectal crypt, faithfully reflecting normal epithelial development.^43^ We identified gene expression and regulatory programs defining absorptive and secretory lineages and highlighted *POU2F3* as a key transcriptional regulator of tuft cells from both the transcriptomic and epigenomic perspectives.

In contrast, T‐PDOs displayed marked inter‐patient heterogeneity at both the transcriptomic and epigenomic levels. Using NMF analysis, we identified four distinct transcriptional MPs, including proliferation, stemness, metabolic adaptation, and an unclassified program. The relative abundance of these programs varied among patients, reinforcing the concept of patient‐specific transcriptional ecosystems within CRC. Despite this variability, a consistent stem‐like transcriptional program was observed across T‐PDOs, highlighting a shared cellular state associated with tumor development and resistance to differentiation. Notably, these tumor states were also reflected in predicted cell type compositions.

Our genomic analysis revealed that T‐PDOs retained key driver mutations found in the primary tumor but also acquired de novo mutations absent in both the primary tissue and N‐PDOs. This suggests the ongoing genomic evolution and clonal selection occur in organoid cultures, potentially driven by the selective expansion of specific tumor subclones. Functionally, T‐PDOs harboring *KRAS* mutations exhibited differential responses to targeted therapies, highlighting mutation‐specific vulnerabilities and confirming the relevance of our genomic findings.

A small proportion of Cancer1 cells was detected in the normal tissue samples. This may reflect minor tumor contamination during sampling or early molecular changes in tumor‐adjacent epithelium. Our WXS analysis reflects the minimal infiltration of tumor cells in the adjacent normal samples (Table S14). Supporting the latter, Cancer1 cells were enriched for inflammation‐related pathways, suggesting a potential pre‐neoplastic or transitional state contributing to their transcriptomic similarity and co‐clustering with tumor cells.

We also interrogated fibroblast‐epithelial interactions within the TME. *FAP*‐expressing CAFs exhibited distinct transcriptional programs enriched in EMT and angiogenesis‐related pathways. Ligand‐receptor analyses revealed activin signaling as a potentially unique fibroblast‐tumor axis in CRC. Given *INHBA*'s known roles in promoting EMT in breast cancer^48^ and being associated with CRC progression^49^ and prognosis,^50^ these findings suggest that CAFs drive tumor progression via *INHBA*‐*ACVR1B*/*ACVR2A* and downstream TGF‐beta/SMAD4 pathways.

Elevated *HIF1A* expression in CAFs suggested a role in matrix remodeling^33^, ^34^ and hypoxia‐driven tumor cell migration. These data point to possible functionally specialized stromal populations that share the tumor niche and may influence therapy resistance.

In summary, our multi‐omics atlas of primary CRC tissue and matched PDOs demonstrates that while N‐PDOs model homeostatic epithelial biology, T‐PDOs retain core molecular features of malignancy, including somatic mutations and stem‐like transcriptional programs. However, they also evolve distinct features, both genetically and transcriptionally, over time in culture. These findings highlight the utility of PDOs in capturing both conserved and patient‐specific aspects of CRC biology, while also cautioning that clonal dynamics and culture‐driven adaptation may alter tumor heterogeneity. Importantly, the consistent presence of a stem‐like program across T‐PDOs emphasizes the relevance of this cell state in CRC pathogenesis and suggests its potential as a prognostic marker or therapeutic target. Moving forward, integrating PDO‐based profiling into clinical pipelines could aid in patient stratification, drug response prediction, and the development of personalized treatment strategies for CRC.

## AUTHOR CONTRIBUTIONS

**Zhijun Yu:** Conceptualization; writing – original draft; writing – review and editing; visualization; data curation; software; validation; formal analysis; investigation. **Merel Derksen:** Methodology; writing – review and editing; data curation. **Brigit M. te Pas:** Methodology; writing – review and editing. **Sabrina Ladstätter:** Writing – review and editing; methodology; data curation. **Rene Overmeer:** Methodology; validation; writing – review and editing. **Peter Brazda:** Software; writing – review and editing. **Marc van de Wetering:** Writing – review and editing; investigation. **Farzin Pourfarzad:** Methodology; writing – review and editing; data curation. **Robert G. J. Vries:** Methodology; writing – review and editing; resources. **Wout Megchelenbrink:** Software; writing – review and editing; data curation. **Christoph Bock:** Conceptualization; funding acquisition; writing – review and editing; resources. **Lucia Altucci:** Conceptualization; supervision; writing – review and editing; formal analysis; investigation; funding acquisition. **Hendrik G. Stunnenberg:** Conceptualization; supervision; funding acquisition; project administration; writing – original draft; writing – review and editing; resources; formal analysis; investigation.

## CONFLICT OF INTEREST STATEMENT

Christoph Bock is a cofounder and scientific advisor of Myllia Biotechnology and Neurolentech. Other authors declare no competing interests.

## ETHICS STATEMENT

All patient material was collected under the approval of the medical ethics review committee (medisch ethische toetsings commissie [METC]) and the Biobanken review committee (toetsingscommissie biobanken [TcBio]). Patients provided informed consent under the HUB‐Cancer protocol (12‐093). The use of the organoids in the HCA Organoid project was approved by TcBio under release protocol #20‐702 and previous proof‐of‐concept studies #19‐586, #19‐797, and #19‐799 (Foundation Hubrecht Organoid Biobank; https://www.hubrechtorganoidbiobank.org/).

## Supporting information


**Data S1.** Supporting Information.

## Data Availability

The sequence data generated in this study regarding the personally identifiable data (fastq files or bam files) was submitted to The European Genome‐Phenome Archive (EGA) under controlled access by the HCA|Organoid cohort; the accession numbers for this study are EGAS00001008067 and EGAS00001008110. snRNA‐seq and snATAC‐seq were submitted to the public repository of Gene Expression Omnibus (GEO) with the accession number GSE294559. The code for analyzing could be found: https://github.com/zhijunyuu/CRC. Further information is available from the corresponding author upon request.
