# Supplementary material for: Single‐cell multi‐omics characterize colorectal tumors, adjacent healthy tissue and matched (tumor) organoids identifying CRC‐unique features
Source: Int J Cancer. 2025 Aug 23;157(12):2619–34. doi: 10.1002/ijc.70103 (PMC12541563; doi:10.1002/ijc.70103)
Supplement: Supplementary file 1 — Data S1. Supporting Information. [file IJC-157-2619-s001.pdf]

Single-cell multi-omics characterize colorectal tumors, adjacent healthy tissue and matched organoids identifying CRC-unique features

Zhijun Yu, Merel Derksen, Brigit M. te Pas, Sabrina Ladstätter, Rene Overmeer, Peter Brazda, Marc van de Wetering, Farzin Pourfarzad, Robert G J Vries, Wout Megchelenbrink, Christoph Bock, Lucia Altucci, Hendrik G Stunnenberg

**Table of contents**	
**Supplementary Methods**	**Page 2-10**
**Supplementary Tables**	**Page 11-48**
**Supplementary Figures**	**Page 49-55**
**Supplementary References**	**Page 56**

Supplementary Methods

Primary tissue sample material

All nine-patient material was collected under the approval of the medical ethics review committee (medisch ethische toetsings commissie (METC)) and the Biobanken review committee (toetsingscommissie biobanken (TcBio)). Patients provided informed consent under the HUB-Cancer protocol (12-093). Primary tissue samples were collected during surgical procedures, including both tumor and adjacent normal tissue (at least 5 cm away from the tumor site). For the adjacent normal tissue, visible fatty and muscle layers were removed prior to digestion. Subsequently, the tissue was dissociated using collagenase type II, producing both intact crypts (when available) and single cell suspensions, which were separated by filtering and cryopreserved separately following collagenase digestion. The duration of digestion varied from each sample, ranging from 60 to 140 minutes. Tumor tissue single-cell suspensions were obtained after collagenase digestion and cryopreserved. The primary tissue samples were collected, processed and cryopreserved by HUB Organoids B.V. (HUB). After a favorable approval of this project by the Biobanken review committee, the cryopreserved samples were then transferred on dry ice to Princess Maxima Center for Pediatric Oncology (PMC) and stored in liquid nitrogen until needed.

Organoid material

For organoid differentiation, the organoids were initially cultured in their respective expansion medium starting from day 0. Between day 3 and day 7, a combination colon differentiation medium (cCDM**, Table S2**[1]) was employed before harvesting and counting the organoids. Intact organoids were then collected and cryopreserved in freezing medium. The generation, culturing and preparation of patient-derived organoids were conducted by HUB. All prepared organoid samples were subsequently transferred to PMC on dry ice and stored in liquid nitrogen prior to use. All the

patient derived normal and tumor organoids (PDOs) are available and can be obtained from Foundation Hubrecht Organoid Biobank (https://www.hubrechtorganoidbiobank.org/).

Whole-exome sequencing

Genomic DNA was isolated using a Qiagen QiaSymphony SP. The DNA quantity and quality was measured by PicoGreen and agarose gel electrophoresis. Exome capture libraries were generated using 1ug of input gDNA and the Agilent SureSelectXT Low Input Target Enrichment protocol for Illumina paired-end sequencing library. 250 ng of DNA library was mixed with hybridization buffers, blocking mixes, RNase block and 5 µl of SureSelect all exon capture library, according to the standard Agilent SureSelect Target Enrichment protocol. The captured DNA was washed and amplified. Then final purified product was quantified by qPCR according to the qPCR Quantification Protocol Guide (KAPA Library Quantification kits for Illumina Sequencing platforms) and qualified by the TapeStation DNA screen tape D1000 (Agilent). Libraries were sequenced on an illuminated NovaSeq6000 platform. Adjacent normal and normal organoids were used as germline control. Somatic variant calling was performed with the Genome Analysis Toolkit 4 (GATK4).

Oxford Nanopore Technologies sequencing

cDNA libraries from 10x Multiome experiments were used with the ligation sequencing V14 protocol (SQK-LSK114) to prepare libraries for Oxford Nanopore PreomethION. Passed QC FASTQ files were processed with the epi2me-labs/wf-single-cell pipeline (https://github.com/epi2me-labs/wf-single-cell). UMIs supporting the REF and ALT alleles for known somatic variants (WXS) were counted for each cell using the VarTrix toolkit (https://github.com/10XGenomics/vartrix).

Drug screening

For the screening assays, organoids were passaged 1-2 days in advance and seeded at high density to obtain organoids with average size between 20 to 100 µm. Subsequently organoids were harvested by adding Dispase (Sigma) at final concentration of 1 mg/mL and incubating at 37°C for 30 min to 1 hour to digest Matrigel. The organoids were filtered between 20 and 100 µm and counted. After counting, 250 organoids were dispensed per screening well in a total volume of 40 µL of organoid expansion medium (with 5% Matrigel) in ultra-low attachment (ULA) 384 well plates. Compounds were dispensed 1-3 hours after seeding the plates. All wells were normalized to highest DMSO concentration (with a max final concentration no higher than 1%). Organoids were exposed to several dilution series and incubated for 5 days without further medium or compound refreshment. Viability was measured by an ATP-based (Cell-Titer-Glo® 3D; Promega, G9683).

Single-nucleus suspension preparation

Cryopreserved primary samples and organoid samples were thawed in a 37°C water bath following standard thawing protocols. Next, the cell pellet was resuspended in NP40 Lysis Buffer (**Table S3**) and transferred to a 70 µm MACS SmartStrainer (Miltenyi Biotec) tissue grinder. To isolated nuclei from the tissue, the sample was processed with five strokes using a loose pestle, followed by ten strokes with a tight pestle. Then, the nuclei suspension went through 70 µm and 40 µm Flowmi cell strainer (Bel-Art), followed by 7-AAD staining (1:1000, Invitrogen 15239004) before sorting. Fluorescence-activated nucleus sorting (FANS) was used to purify and collect intact nuclei, with at least 200,000 nuclei collected from each sample. For the nuclei permeabilization step, 0.1x Lysis Buffer (**Table S4-6**) was used, and the nuclei were suspended in Diluted Nuclei Buffer (**Table S7**) to achieve a final concentration of around 5000 nuclei/µl. All buffers used in the protocol were freshly prepared.

Single-nucleus RNA and ATAC libraries preparation and sequencing

snRNA and snATAC libraries were prepared according to the protocol provided by the vender using the 10x Genomics Chromium Next GEM Single Cell Multiome ATAC + Gene Expression kit (CG000338 RevE). In brief, transposition was performed on nuclei, followed by the formation of functional Gel Beads in Emulsion (GEMs), which were also barcoded. This process was followed by post-GEM incubation cleanup, pre-amplification PCR, ATAC library construction, cDNA amplification and gene expression library construction. As a result, fragments from the same single nucleus carry the same barcode, enabling sequencing reads to be mapped back to the same original nucleus. Every snRNA and snATAC library were quality-checked using the Agilent Bioanalyzer High Sensitivity DNA chip on a 2100 Bioanalyzer before sequencing. The libraries were sequenced on the Illumina NovaSeq 6000 platform at PMC, using a paired-end and dual indexing setup as recommended from 10x Genomics. The sequencing parameters were as follows: for snRNA libraries: Read 1: 28 bp, i7 Index: 10 bp, i5 Index: 10bp, Read 2: 90 bp; and for snATAC libraries: Read 1N: 50 bp, i7 Index: 8 bp, i5 Index: 24 bp, Read 2N: 50 bp. Approximately 50,000 reads per nucleus were expected for both snRNA and snATAC libraries.

Pre-processing single-nucleus RNA and ATAC sequencing data

The Cell Ranger ARC 2.0.2 pipelines (from 10x Genomics) were used to process the multiomics sequencing data. The pipelines first demultiplexed the BCL files into FASTQ files, which were then converted into BAM files and feature-barcode matrixes. The pipeline performed alignment to the UCSC hg38 transcriptome, barcode counting, UMI counting/peak calling and ultimately produced feature-barcode matrixes as output. A multiplexing strategy was used in this project, where samples from two individual patients were pooled prior to library preparation within a 10x Genomics Multiome kit reaction. This strategy leverages natural genetic variation, allowing computational tools to demultiplex samples based on those variations, such as single nucleotide

polymorphisms (SNP). Specifically, the software tools cellsnp-lite[2] and vireo[3] were used for the SNP-based demultiplexing, assigning 10x barcodes to different patients. SNP files were generated independently by cellsnp-lite for the snRNA-seq and snATAC-seq libraries. These SNP files were then concatenated into a matrix, and vireo was used to separate the samples by patients. In addition to SNP variation information, gender information also utilized during the demultiplexing process to further distinguish between the two pooled patients.

snRNA-seq data processing

1) Integration, clustering, cell type annotation

CellBender[4] was used to generated ambient RNA corrected count matrices, and doublets were identified and excluded using DoubletFinder[5]. The count matrix from each library was merged and filtered using the following criteria: 500 < gene count < 10,000, UMI counts > 500, and percentage of mitochondria < 20%. The merged data was subsequently processed using Seurat (v5.0.1)[6]. Briefly, data normalization was performed using SCTransform, followed by principal component analysis (PCA) analysis with RunPCA. The FindNeighbor function was used to compute the 40 nearest neighbors (k=40), clustering was conducted using the Leiden algorithm, and the data was projected onto a two-dimension plot using RunUMAP. Cell type annotation was performed manually and based on classical cell type markers.

2) Cell type proportion

For each cell type, the proportion of that cell type per patient was calculated and compared between the normal and tumor groups. The p-values were determined using a Wilcox.test to assess the statistical significance of differences between the normal and tumor groups.

3) DEG, GO and GSEA

Differentially expressed genes (DEGs) for each cell type were identified using the FindAllMarkers function in Seurat[6], with parameter ‘min.pct=0.1’ and ‘logfc.threshold=0.25’. These DEGs were then used as input for Gene Ontology (GO) enrichment analyze using the

clusterProfiler [7] package with default settings. Additionally, the fgsea and msigdbr[8] packages were employed to analyze Gene Set Enrichment Analysis (GSEA) of Hallmark pathways for the epithelial and fibroblast compartments.

4) NMF

To identify the transcriptional programs commonly active across tumor PDOs, the RunNMF function from the swne[9] package was applied to the snRNA-seq data from each patient. This function performed non-negative matric factorization (NMF) with k=15, resulting in 135 NMF programs (9 patients x 15 programs per patient). The top 50 genes from each NMF program were selected as input for the AddModuleScore function*.* The resulting enrichment score matrix was then used to compute Pearson correlation and generate a heatmap. Hierarchical clustering of the Pearson correlation and top genes identified four meta-programs (MPs). Finally, GOs analysis was performed on these MPs for annotation purposes.

5) Pseudotime trajectory analysis

Monocle3[10] was used to analyze the developmental trajectory and pseudotime of colorectal normal PDOs based on the snRNA-seq data. The “Stem cells” cluster was designated as the starting point for the trajectory. For plotting gene expression along the pseudotime trajectory, clusters related to the absorptive or secretory lineage were selected respectively.

6) Cell-cell interaction analysis

Cellchat[11] was used to analyze cell-cell interactions by detecting ligand-receptor interactions in the snRNA-seq data using the standard pipeline. The count matrix and cell type annotation were used as input for the creation of CellChat objects. CellChat objects were generated for normal and tumor samples, independently. The cell-cell communication networks pathways were compared between normal and tumor samples.

7) iCNV analysis

Inferred copy number variation (iCNV) was calculated using the Numbat[12] package based on snRNA-seq data. For per patient, the primary normal tissue data was used as reference to

determine the iCNV in the matched primary tumor tissue data. The output from Numbat, which includes single-cell clone assignment, was then used as input to generate the iCNV plot on UMAP.

8) Anchor-based cell type prediction

To compare primary epithelial cell states with those epithelial cells derived organoid, ancho-based cell type prediction was employed. The organoid data served as the query data, while the primary normal epithelial data acted as the reference. Initially, anchors were identified using the FindTransferAnchor function from Seurat[13]. Next, the Mapquery function was used to project the query onto the reference UMAP, enabling the projection of predicted cell type annotations onto the query data.

9) snRNA-seq data based MSI detection

The MSI/MSS status of primary tumor tissue was determined using snRNA-seq data. The count matrix from each patient was used as input, and the TCGA.CRC.model.pkl model from MSIsensor-RNA[14] was employed for analysis. The Msisensor-rna detection function was used to predict the MSI/MSS state of the data.

10) Gene signature score and spatial analysis

Normalized gene expression data were used to compute average expression scores for HLA class I (*HLA-A, HLA-B, HLA-C*) and CD8+ T cell (*CD2, CD3D, CD8A, CD27, CD28*) gene signature in each spatial spot using Seurat[13]. Pearson correlation was calculated between the two scores across spots.

snATAC-seq data processing

1) Integration, clustering and peak calling

The count matrix from each library was merged and filtered with the following criteria: 3,000 < Tn5 cut sites < 120,000, TSS.enrichment > 3 and nucleosome_signal < 3. The merged data was then processed using standard procedures from the Signac package (v1.12.0)[15]. In brief,

data normalization was performed using the RunTFIDF() function, then feature selection was conducted with the FindTopFeatures () function. Next, dimension reduction was achieved using RunSVD() function. Finally, non-linear dimensionality reduction and clustering were carried out with the RunUMAP(), FindNeishbors() and FindClusters() functions. Peak calling was performed with the MACS2 package[16] and subsequent analyses were based on the results from the peak calling ("peak" assay).

2) DARs analysis

Differential accessible regions (DARs)[17] were identified using the FindAllMarkers() function[15], with parameters 'logfc.threshold=0.1', 'min.pct=0.2' and 'test.use="LR"'. Regions with an average log2 fold change (avg_log2FC) greater than 1 were selected as input for AverageExpression() function. Finally, a heatmap of these regions was plotted using the pheatmap package (v.1.0.12).

3) Motif analysis and closest gene

The results from MACS2 peaks calling (peaks assay) were used to perform motif analysis with the AddMotifs() and RunChromVAR() functions in Signac. These functions assessed motif activities deviations in chromatin accessibility across peak regions, creating chromvar assay in Seurat object. The cell type specific accessible regions were used as input to get the cell type specifically enriched TF motifs using FindMotif() function, and to get the closest feature to a given set of genomic regions using ClosestFeature() function.

Statistical analysis

No statistical method was used to predetermine the sample size. The experiments were not randomized, and the investigators were not blinded to allocation during library preparation or analysis. Differentially expressed genes for the snRNA-seq data and differentially accessible regions for the snATAC-seq data were assessed for statistical significance using the Wilcoxon Rank sum test. Differences in the cell type proportions between normal and tumor conditions were

analyzed by the Wilcoxon Rank sum test. In the box plots, the central lines represent the median, the box boundaries indicate the upper and lower quartiles, and the whiskers indicate 1.5x the interquartile range. The Benjamini-Hochberg (BH) controlled False Discovery Rate (FDR) was used to define the enrichment significance for the gene oncology analysis. A p-value of less than 0.05 was considered statistically significant. Statistical analyses were performed using R and Python.

Supplementary Tables

Supplementary table1

Table S1 Patient meta-data							
**Patient number**	**Patient ID**	**Gender**	**Age**	**Tumor location**	**Pre-treatment**	**Pathology WHO**	**TNM**
Patient1	P001	F	55	Rectum	No	adenocarcinoma	pT3N0
Patient2	P002	M	76	Sigmoid	No	adenocarcinoma	pT3N0
Patient3	P003	M	74	Ascending colon	Chemotherapy:2x CAPOX	adenocarcinoma	pT3N1b
Patient4	P004	M	74	Rectum	No	adenocarcinoma	pT2N0
Patient5	P005	F	59	Ascending colon	No	adenocarcinoma	pT3N0
Patient6	P006	F	89	Cecum	No	Medullary adenocarcinoma	pT4aN1a
Patient7	P007	M	75	Rectum	No	adenocarcinoma	pT2N0
Patient8	P008	M	56	Rectum	No	adenocarcinoma	pT2N0
Patient9	P009	M	63	Sigmoid	No	adenocarcinoma	pT1N0

This table presents the metadata of patients, as listed patient number, gender, age, tumor location and details regarding the formation of the primary normal samples.

Supplementary table2

Table S2 Expansion and differentiation media					
**Reagent**	**CNM (WNT surrogate)**	**CNM**	**cCDM**	**CTM**	**Vender**
**Advanced DMEM/F12**	44.50%	44.50%	45%	44.50%	Gibco
**GlutaMax**	1 mM	1 mM	1 mM	1 mM	Gibco
**HEPES**	5 mM	5 mM	5 mM	5 mM	Gibco
**Pen/strep**	0.25%	0.25%	0.25%	0.25%	Gibco
**N-Acetylcysteine**	1.25 mM	1.25 mM	1.25 mM	1.25 mM	Sigma-Aldrich
**A83-01**	500 nM	500 nM	500 nM	500 nM	Bio-Techne
**B27 Supplement**	1X	1X	1X	1X	Gibco
**EGF**	50 ng/ml	50 ng/ml	50 ng/ml	50 ng/ml	Pepro Tech
**Gastrin**	5 nM	5 nM	5 nM	5 nM	Bio-Techne
**Noggin**	100 ng/ml	100 ng/ml	100 ng/ml	100 ng/ml	Pepro Tech

**Nicotinamide**	10 mM	10 mM	-	10 mM	Sigma-Aldrich
**Primocin**	50 μg/ml	50 μg/ml	50 μg/ml	50 μg/ml	Invitrogen
**Rspondin 3**	250 ng/ml	250 ng/ml	250 ng/ml	250 ng/ml	R&D
**DAPT**	-	-	10 μM	-	Sigma-Aldrich
**SB202190**	10 μM	10 μM	-	10 μM	Sigma-Aldrich
**PD0325901**	-	-	100 nM	-	Sigma-Aldrich
**Wnt surrogate**	0.5 nM	-	-	-	UPE

Supplementary table3

Table S3 NP40 Lysis Buffer		
**NP40 Lysis Buffer**	**Final concentration**	**Vender**
Tris-HCl (pH7.4)	10 mM	Sigma-Aldrich
NaCl	10 mM	VWR
MgCl2	3 mM	Sigma-Aldrich
Nonidet P40 Subseitute	0.10%	Sigma-Aldrich
DTT	1 mM	Sigma-Aldrich
Rnase inhibitor	1 U/ µL	Roche

Supplementary table4

Table S4 1XLysis Buffer		
**1XLysis Buffer**	**Final concentration**	**Vender**
Tris-HCl (pH7.4)	10 mM	Sigma-Aldrich
NaCl	10 mM	VWR
MgCl2	3 mM	Sigma-Aldrich
Tween-20	0.10%	ThermoFisher
Nonidet P40 Subseitute	0.10%	Sigma-Aldrich
Digitonin	0.01%	Invitrogen
BSA	0.10%	ThermoFisher
DTT	1 mM	Sigma-Aldrich
Rnase inhibitor	1 U/ µL	Roche

Supplementary table5

Table S5 Lysis Dilution Buffer		
**Lysis Dilution Buffer**	**Final concentration**	**Vender**
Tris-HCl (pH7.4)	10 mM	Sigma-Aldrich
NaCl	10 mM	VWR
MgCl2	3 mM	Sigma-Aldrich
BSA	1%	ThermoFisher
DTT	1 mM	Sigma-Aldrich
Rnase inhibitor	1 U/ μL	Roche

Supplementary table6

Table S6 0.1X Lysis Buffer	
**0.1XLysis buffer**	**Final concentration**
1X Lysis Buffer	0.1X
Lysis Dilution Buffer	-

Supplementary table7

Table S7 Diluted Nuclei Buffer		
**Diluted Nuclei Buffer**	**Final concentration**	**Vender**
Nuclei Buffer (20X)	1X	10X Genomics
DTT	1 mM	Sigma-Aldrich
Rnase inhibitor	1 U/ μL	Sigma-Aldrich

Supplementary table8

Table S8 WXS QC								
**Tissue/Organoid**	**Normal/Tumor**	**Patient ID**	**Library**	**Total number of sequenced reads**	**Total number of uniquely mapped non-duplicate reads**	**Total number of covered bases**	**Median coverage (range) per base**	**Percentage of targeted bases ≥10X (%)**
Tissue	Normal	P001	NP001	37567653	37504231	35602238	48.28 (0.0-857.21)	98.68
Organoid	Normal	P001	NO001	42139743	42079558	35573801	53.03 (0.0-954.14)	98.06
Organoid	Tumor	P001	TO001	42854863	42781193	35567402	52.55 (0.0-1076.6)	98.03
Tissue	Normal	P002	NP002	44502360	44427090	35681503	58.0 (0.0-1026.61)	99.23
Organoid	Normal	P002	NO002	42673437	42605725	35643985	53.59 (0.0-1033.57)	98.35
Tissue	Tumor	P002	TP002	42241655	42182994	35663263	54.27 (0.0-1049.77)	98.84

Organoid	Tumor	P002	TO002	39917759	39844241	35585520	47.69 (0.0-1080.92)	97.86
Tissue	Normal	P003	NP003	44879339	44794360	35683995	59.58 (0.0-1209.07)	99.34
Organoid	Normal	P003	NO003	67034827	66894475	35688162	88.06 (0.0-2029.02)	99.42
Tissue	Tumor	P003	TP003	42590668	42509663	35674017	55.42 (0.0-993.89)	99.17
Organoid	Tumor	P003	TO003	45300186	45224960	35666804	58.7 (0.0-1098.14)	99.06
Tissue	Normal	P004	NP004	41104368	41048747	35669020	52.83 (0.0-1294.98)	98.91
Organoid	Normal	P004	NO004	47064613	46973016	35673627	61.19 (0.0-1335.3)	99.02
Tissue	Tumor	P004	TP004	40681300	40619159	35671733	51.67 (0.0-1315.53)	98.9
Organoid	Tumor	P004	TO004	42693831	42597315	35647732	54.6 (0.0-1177.9)	98.55

Tissue	Normal	P005	NP005	43907919	43821085	35616018	58.3 (0.0-1103.03)	99.13
Organoid	Normal	P005	NO005	40025521	39947552	35612270	53.66 (0.0-979.42)	99.02
Tissue	Tumor	P005	TP005	44097846	44020582	35616593	58.63 (0.0-1152.05)	99.15
Organoid	Tumor	P005	TO005	72252610	72124489	35627396	92.61 (0.0-1945.65)	99.38
Organoid	Normal	P006	NO006	38181273	38124243	35581017	49.31 (0.0-1419.34)	98.2
Organoid	Tumor	P007	TO007	49876177	49805904	35643128	64.74 (0.0-1165.72)	98.75
Organoid	Tumor	P008	TO008	39476083	39388124	35654247	45.28 (0.0-1674.28)	98.35
Organoid	Normal	P008	NO008	29758862	29645609	35638190	38.41 (0.0-917.51)	97.85
*Reference genome: GRCh38								

*N: normal, T: tumor, P: primary, O: organoid.								

Supplementary table9

Table S9 ONT QC									
**Sample ID**	**Total number of sequenced reads**	**Total number of uniquely mapped non-duplicate reads**	**RNA integrity number (RIN)**	**Ratio of all reads aligned to rRNA regions to total uniquely mapped reads (rRNA rate)**	**Ratio of exon-mapped reads to total uniquely mapped reads (Expression profile efficiency)**	**Total number of detected transcripts with reads > =1**	**Median read length**	**N50 length**	**Median read quality**
TO001_TO002	121.63 M	85585462	not applicable	0.02	35.80%	68990107	906	1.09 kb	16
TO006_TO007	128.49 M	84629968	not applicable	0.08	35.40%	67003725	852	1.01 kb	15
*Reference genome: GRCh38									
*N: normal, T: tumor, P: primary, O: organoid.									

Supplementary table10

Table S10 Multiome-snRNA-seq QC							
**Sample ID**	**Library ID**	**Total number of sequenced reads per library**	**Total number of uniquely mapped reads per library**	**Total number of called cells**	**Median number (and range) of uniquely mapped reads per called cell**	**Median rRNA rate (and range) per called cell**	**Median number (and range) of detected genes per called cell**
NP001_snRNA	NP001_NP002	583207352	497009305	10958	19753 (155 - 1467579)	0.178 (0.078 - 0.913)	2434 (48 - 17731)
NP002_snRNA	NP001_NP002	583207352	497009305	10958	19753 (155 - 1467579)	0.178 (0.078 - 0.913)	2434 (48 - 17731)
NP003_snRNA	NP003_NP056	252644148	228213459	5118	26647 (188 - 1498339)	0.127 (0.046 - 0.921)	1951 (27 - 14078)
NP004_snRNA	NP051_NP004	378901045	342450764	7904	19537.5 (160 - 2593779)	0.135 (0.058 - 0.927)	2276.5 (33 - 17985)
NP005_snRNA	NP005_NP054	409787814	298038677	8345	22048 (301 - 991296)	0.315 (0.069 - 0.953)	1455 (42 - 9936)
NP006_snRNA	NP048_NP006	287915152	214669537	5610	21006.5 (398 - 878339)	0.279 (0.089 - 0.956)	2223 (74 - 16168)

NP007_snRNA	NP007_NP060	287084145	228174478	5013	23109 (227 - 1880531)	0.188 (0.072 - 0.982)	2474 (42 - 17476)
NP008_snRNA	NP008_NP067	383343349	277693922	7193	29336 (310 - 861464)	0.292 (0.069 - 0.978)	2320 (34 - 14765)
NP009_snRNA	NP070_NP009	695983065	602721334	13635	30636 (290 - 1190055)	0.143 (0.050 - 0.954)	2371 (36 - 16275)
TP001_snRNA	TP001_TP002	650092185	470146668	15313	12952 (151 - 854798)	0.277 (0.082 - 0.963)	1386 (25 - 11356)
TP002_snRNA	TP001_TP002	650092185	470146668	15313	12952 (151 - 854798)	0.277 (0.082 - 0.963)	1386 (25 - 11356)
TP003_snRNA	TP003_TP008	296998130	216422537	6040	19216.5 (217 - 920157)	0.283 (0.077 - 0.986)	2109.5 (47 - 11985)
TP004_snRNA	TP004_TP005	385672612	249761584	8421	16303 (346 - 737376)	0.385 (0.078 - 0.954)	1572 (57 - 12134)
TP005_snRNA	TP004_TP005	385672612	249761584	8421	16303 (346 - 737376)	0.385 (0.078 - 0.954)	1572 (57 - 12134)

TP006_snRNA	TP006_TP007	371856006	289638643	8002	18990.5 (332 - 651223)	0.199 (0.059 - 0.968)	2249 (69 - 14226)
TP007_snRNA	TP006_TP007	371856006	289638643	8002	18990.5 (332 - 651223)	0.199 (0.059 - 0.968)	2249 (69 - 14226)
TP008_snRNA	TP003_TP008	296998130	216422537	6040	19216.5 (217 - 920157)	0.283 (0.077 - 0.986)	2109.5 (47 - 11985)
TP009_snRNA	TP009	511909380	380655815	9373	19240 (495 - 672930)	0.259 (0.058 - 0.927)	1539 (58 - 12719)
NO001_snRNA	NO001_NO002	150803234	139191385	3730	17799 (65 - 2219247)	0.097 (0.035 - 0.888)	2423.5 (16 - 17341)
NO002_snRNA	NO001_NO002	150803234	139191385	3730	17799 (65 - 2219247)	0.097 (0.035 - 0.888)	2423.5 (16 - 17341)
NO003_snRNA	NO003_NO051	492274848	450037666	9395	34651 (327 - 1689929)	0.105 (0.058 - 0.960)	3164 (27 - 15264)
NO004_snRNA	NO006_NO004	657151850	609574056	13311	38927 (168 - 1412207)	0.103 (0.064 - 0.918)	3293 (32 - 14649)

NO005_snRNA	NO052_NO005	543179488	502712616	11376	31687 (106 - 2876931)	0.097 (0.035 - 0.948)	3258.5 (28 - 17230)
NO006_snRNA	NO006_NO004	657151850	609574056	13311	38927 (168 - 1412207)	0.103 (0.064 - 0.918)	3293 (32 - 14649)
NO007_snRNA	NO007_NO060	658348705	607195011	13671	33746 (77 - 1656364)	0.101 (0.044 - 0.911)	3136 (19 - 15714)
NO008_snRNA	NO045_NO008	479110918	440973689	11454	26055 (166 - 2714221)	0.107 (0.043 - 0.975)	2800 (22 - 17201)
NO009_snRNA	NO017_NO009	621657796	577271429	16190	31634.5 (89 - 795734)	0.096 (0.047 - 0.896)	3542.5 (23 - 12705)
TO001_snRNA	TO001_TO002	384254172	359661905	10118	17459 (81 - 1147605)	0.119 (0.051 - 0.729)	2972 (32 - 16818)
TO002_snRNA	TO001_TO002	384254172	359661905	10118	17459 (81 - 1147605)	0.119 (0.051 - 0.729)	2972 (32 - 16818)
TO003_snRNA	TO020_TO003	458402027	425442921	5959	24886 (232 - 1795146)	0.132 (0.072 - 0.922)	3661 (40 - 17998)

TO004_snRNA	TO004_TO016	538066627	493084257	14848	20851 (186 - 867730)	0.123 (0.053 - 0.929)	2951 (43 - 14819)
TO005_snRNA	TO005	501829342	461381897	8266	42429 (209 - 1667351)	0.126 (0.040 - 0.871)	3436.5 (42 - 14356)
TO006_snRNA	TO006_TO007	503329708	457577038	11582	25422.5 (272 - 1254468)	0.117 (0.050 - 0.946)	3363 (44 - 15844)
TO007_snRNA	TO006_TO007	503329708	457577038	11582	25422.5 (272 - 1254468)	0.117 (0.050 - 0.946)	3363 (44 - 15844)
TO008_snRNA	TO017_TO008	411402319	379600920	8290	26440 (193 - 1230182)	0.121 (0.062 - 0.976)	3676 (40 - 16853)
TO009_snRNA	TO009	359953401	337060365	11920	16069 (76 - 1006356)	0.120 (0.041 - 0.578)	2826 (26 - 15819)
*Reference genome: GRCh38							
*Multiplexing: some of the libraries include two samples, here in the table represents data from per library.							

*N: normal, T: tumor, P: primary, O: organoid.							

Supplementary table11

Table S11 Multiome-snATAC-seq QC					
**Sample ID**	**Library ID**	**Total number of sequenced reads per library**	**Median number (and range) of uniquely mapped reads per called cell**	**Total number of called cells**	**Median fraction (and range) of reads in regions of interest per called cell**
NP001_snATAC	NP001_NP002	430655713	7974 (10 - 449036)	10958	0.344 (0.209 - 0.739)
NP002_snATAC	NP001_NP002	430655713	7974 (10 - 449036)	10958	0.344 (0.209 - 0.739)
NP003_snATAC	NP003_NP056	581427351	25806.5 (38 - 1657674)	5118	0.626 (0.223 - 0.812)
NP004_snATAC	NP051_NP004	425015853	11733 (37 - 1142233)	7904	0.658 (0.219 - 0.829)
NP005_snATAC	NP005_NP054	406509739	9733 (16 - 779539)	8345	0.601 (0.186 - 0.863)
NP006_snATAC	NP048_NP006	250953039	4239.5 (6 - 362453)	5610	0.478 (0.133 - 0.741)
NP007_snATAC	NP007_NP060	247547632	9621 (14 - 610923)	5013	0.487 (0.174 - 0.773)
NP008_snATAC	NP008_NP067	355318161	8911 (12 - 546924)	7193	0.462 (0.159 - 0.765)
NP009_snATAC	NP070_NP009	735610244	12478 (17 - 1035448)	13635	0.613 (0.202 - 0.836)
TP001_snATAC	TP001_TP002	548911265	8565 (34 - 681242)	15313	0.397 (0.177 - 0.808)
TP002_snATAC	TP001_TP002	548911265	8565 (34 - 681242)	15313	0.397 (0.177 - 0.808)
TP003_snATAC	TP003_TP008	283041175	9944 (24 - 524383)	6040	0.514 (0.181 - 0.783)
TP004_snATAC	TP004_TP005	361874022	8230 (26 - 206905)	8421	0.343 (0.148 - 0.690)

TP005_snATAC	TP004_TP005	361874022	8230 (26 - 206905)	8421	0.343 (0.148 - 0.690)
TP006_snATAC	TP006_TP007	302813771	7143.5 (5 - 556541)	8002	0.468 (0.165 - 0.763)
TP007_snATAC	TP006_TP007	302813771	7143.5 (5 - 556541)	8002	0.468 (0.165 - 0.763)
TP008_snATAC	TP003_TP008	283041175	9944 (24 - 524383)	6040	0.514 (0.181 - 0.783)
TP009_snATAC	TP009	346023602	6721 (16 - 334123)	9373	0.410 (0.142 - 0.688)
NO001_snATAC	NO001_NO002	180120546	12586.5 (23 - 1151881)	3730	0.713 (0.231 - 0.906)
NO002_snATAC	NO001_NO002	180120546	12586.5 (23 - 1151881)	3730	0.713 (0.231 - 0.906)
NO003_snATAC	NO003_NO051	423440194	16198 (20 - 727534)	9395	0.744 (0.301 - 0.917)
NO004_snATAC	NO006_NO004	691841481	21033 (28 - 706287)	13311	0.690 (0.254 - 0.896)
NO005_snATAC	NO052_NO005	533352875	18984 (31 - 866010)	11376	0.710 (0.280 - 0.876)
NO006_snATAC	NO006_NO004	691841481	21033 (28 - 706287)	13311	0.690 (0.254 - 0.896)
NO007_snATAC	NO007_NO060	874016332	15815 (11 - 832283)	13671	0.691 (0.261 - 0.905)
NO008_snATAC	NO045_NO008	421469577	13833.5 (36 - 936533)	11454	0.700 (0.248 - 0.843)
NO009_snATAC	NO017_NO009	870769176	23117 (44 - 620389)	16190	0.723 (0.285 - 0.867)
TO001_snATAC	TO001_TO002	356570953	14892 (13 - 517578)	10118	0.594 (0.232 - 0.824)
TO002_snATAC	TO001_TO002	356570953	14892 (13 - 517578)	10118	0.594 (0.232 - 0.824)
TO003_snATAC	TO020_TO003	545563671	24261 (28 - 1550082)	5959	0.659 (0.256 - 0.816)
TO004_snATAC	TO004_TO016	470349799	11985 (12 - 549386)	14848	0.632 (0.261 - 0.867)

TO005_snATAC	TO005	435217026	20332 (34 - 626317)	8266	0.616 (0.199 - 0.755)
TO006_snATAC	TO006_TO007	437167225	13777 (12 - 501776)	11582	0.672 (0.245 - 0.851)
TO007_snATAC	TO006_TO007	437167225	13777 (12 - 501776)	11582	0.672 (0.245 - 0.851)
TO008_snATAC	TO017_TO008	507562306	18738.5 (8 - 724078)	8290	0.638 (0.242 - 0.822)
TO009_snATAC	TO009	483094685	17520 (41 - 801382)	11920	0.652 (0.252 - 0.812)
*Reference genome: GRCh38					
*Multiplexing: some of the libraries include two samples, here in the table represents data from per library.					
*N: normal, T: tumor, P: primary, O: organoid.					

Supplementary table12

Table S12 MSIsensor-RNA results			
**Patient ID**	**Number of tumor cells**	**Number of tumor cells predicted as MSS**	**Ratio of MSS cells**
P001	176	176	100%
P002	386	385	99.70%
P003	484	484	100%
P004	79	77	97.40%
P005	1165	1164	99.90%
P006	106	99	93.30%
P007	764	763	99.80%
P008	407	407	100%
P009	57	57	100%

This table lists the results of MSIsensor-RNA, including patient ID, number of tumor cells, number of tumor cells predicted as MSS statue and the ratio of MSS status cells for each patient.

Supplementary table13

Table S13 NMF meta-program (MPs) top 50 genes				
	**MP1**	**MP2**	**MP3**	**MP4**
1	DIAPH3	PLCG2	TRPM3	GMDS
2	BRIP1	EEF1A1	SMOC2	GPHN
3	ATAD2	TPT1	DACH1	MTHFD1L
4	ANLN	MTRNR2L12	ITPR2	ACACA
5	BRCA2	FTH1	CEMIP2	CMSS1
6	POLQ	S100A6	NAALADL2	PUS7
7	ASPM	PTMA	CERS6	WDR43
8	FANCI	CD63	NFAT5	MPP6
9	CIT	TMSB10	KANK1	PUM3
10	NCAPG2	ACSS2	SNTB1	TBC1D4
11	ARHGAP11B	EEF1G	RNF43	FHIT
12	CENPK	GAPDH	NRXN3	CD46
13	HELLS	FLNB	SOX6	FARSB
14	SMC4	CST3	SYK	NDUFAF2
15	MELK	ACSL3	NR6A1	DDX10
16	KIF4A	FAU	ATXN1	ABCC4
17	TPX2	COX6A1	SLC12A2	CAMKMT

18	TOP2A	PRELID2	NKD1	ADK
19	C21orf58	FNDC3B	PROX1	SMYD3
20	KIF14	FTL	PROM1	DDX21
21	KIF11	PPARG	CHRM3	MDN1
22	BARD1	SAMD4A	LRMDA	XPO4
23	XRCC2	PLA2R1	RGMB	PRKDC
24	DTL	SPINT2	ZBTB20	CCSER1
25	KNL1	COX4I1	STARD13	SAMD12
26	KIF18B	MYOF	APBB2	SCFD2
27	RBL1	PPIB	CCSER2	PTPRN2
28	CENPE	CYP3A5	ITGA6	HSPD1
29	CENPF	MIF	TSPAN5	TFF3
30	CDK1	HLA-A	IMMP2L	LRPPRC
31	ATAD5	SGK2	SEMA3C	IARS
32	KIF23	EXT1	SATB2	PLCB4
33	ANXA3	CDC42BPA	UTRN	BZW2
34	PKP4	INPP4B	CAB39L	SND1
35	EZH2	WSB1	VAV3	TSEN2
36	CENPP	UBA52	MME	IMMP2L
37	NUSAP1	TFF3	PATJ	ETS2

38	BRCA1	P4HA1	MGAT5	FRYL
39	GTSE1	DST	TOX3	MAML3
40	RRM2	KRT18	FARP1	VWA8
41	SRGAP1	B2M	PDZRN3	TMTC2
42	DNA2	ABCC3	PTPRN2	MECOM
43	POLA1	MACC1	CRYBG1	ERN2
44	MMS22L	GSTP1	DENND4C	PIP5K1B
45	NDC80	SSR4	PLCG2	SLC4A7
46	KNTC1	TMSB4X	FGGY	TRABD2A
47	E2F7	ERBIN	SEMA3A	CEMIP
48	MKI67	ADAM9	FHIT	RALGAPA2
49	BUB1B	PRAP1	CDK6	TASOR2
50	NUF2	SPAG9	PAM	TRPM3

Supplementary table14

Table S14 REF and ALT counts of TP and TO													
**CHR**	**START**	**END**	**REF**	**ALT**	**FILTER**	**GENE_SYMBOL**	**VARIANT_CLASS**	**dbSNP**	**Tumor_Sample**	**tumor_ref_count**	**tumor_alt_count**	**normal_ref_count**	**normal_alt_count**
chr1	26696860	26696860	C	G	PASS	ARID1A	Missense_Mutation		TO001	12	2	14	0
chr1	233328636	233328636	C	T	PASS	MAP3K21	Missense_Mutation	novel	TO001	38	6	28	0
chr12	25245350	25245350	C	T	PASS	KRAS	Missense_Mutation	rs121913529	TO001	21	48	67	0
chr13	101104635	101104635	C	A	PASS	NALCN	Missense_Mutation	novel	TO001	76	33	73	0
chr17	7674221	7674221	G	A	PASS	TP53	Missense_Mutation	rs121912651	TO001	0	72	147	0
chr2	178554521	178554521	C	T	PASS	TTN	Missense_Mutation	rs758876816	TO001	54	79	105	0

chr3	41233764	41233764	G	A	PASS	CTNNB1	Missense_Mutation	novel	TO001	106	57	148	0
chr1	26697253	26697253	G	-	PASS	ARID1A	Frame_Shift_Del	novel	TO002	12	6	20	0
chr1	228250082	228250082	A	T	PASS	OBSCN	Missense_Mutation	novel	TO002	290	5	356	0
chr1	228279210	228279210	G	A	PASS	OBSCN	Missense_Mutation	rs1161407094	TO002	48	44	178	0
chr1	233346491	233346491	G	T	PASS	MAP3K21	Missense_Mutation	novel	TO002	20	30	71	0
chr12	25245350	25245350	C	T	PASS	KRAS	Missense_Mutation	rs121913529	TO002	22	57	68	0
chr13	101292238	101292238	C	T	PASS	NALCN	Missense_Mutation		TO002	55	117	126	0
chr4	125316968	125316968	G	A	PASS	FAT4	Missense_Mutation		TO002	71	71	183	0
chr5	32712011	32712011	G	C	PASS	NPR3	Missense_Mutation	novel	TO002	27	38	87	0

chr5	11282891 9	11282891 9	C	T	PASS	APC	Nonsense_Mutation	rs13785457 4	TO002	13	11	41	0
chr6	7571983	7571983	G	A	PASS	DSP	Missense_Mutation		TO002	55	41	134	0
chr8	2973156	2973156	G	A	PASS	CSMD1	Missense_Mutation	rs13732956 87	TO002	97	24	70	0
chr1	23335386 8	23335386 8	C	G	PASS	MAP3K21	Missense_Mutation	novel	TO003	48	49	164	0
chr12	25245351	25245351	C	A	PASS	KRAS	Missense_Mutation	rs12191353 0	TO003	31	46	160	0
chr12	56087843	56087843	C	T	PASS	ERBB3	Missense_Mutation	rs14297845 31	TO003	44	54	143	0
chr13	10128395 3	10128395 3	G	A	PASS	NALCN	Missense_Mutation	rs54992695 9	TO003	57	72	90	0
chr17	7673610	7673610	T	C	PASS	TP53	Splice_Site	rs39751643 9	TO003	2	76	189	0

chr2	178584764	178584764	G	A	PASS	TTN	Missense_Mutation	novel	TO003	98	74	300	0
chr2	185801939	185801939	A	T	PASS	FSIP2	Missense_Mutation	novel	TO003	41	50	171	0
chr4	125415615	125415615	G	A	PASS	FAT4	Missense_Mutation		TO003	101	91	347	0
chr4	152326137	152326137	G	C	PASS	FBXW7	Missense_Mutation	rs149680468	TO003	61	57	228	0
chr5	112839515	112839519	AAAAG	-	PASS	APC	Frame_Shift_Del	rs121913224	TO003	0	80	162	0
chr7	21591523	21591523	A	-	PASS	DNAH11	Frame_Shift_Del	novel	TO003	58	26	105	0
chr12	25245348	25245348	C	A	PASS	KRAS	Missense_Mutation	rs121913535	TO004	27	26	69	0
chr17	7674220	7674220	C	T	PASS	TP53	Missense_Mutation	rs11540652	TO004	0	74	166	0
chr18	51065549	51065549	G	A	PASS	SMAD4	Missense_Mutation	rs377767347	TO004	0	66	151	0

chr4	152323074	152323074	C	T	PASS	FBXW7	Missense_Mutation		TO004	63	59	162	0
chr4	152328232	152328232	C	T	PASS	FBXW7	Missense_Mutation	rs1057519895	TO004	70	61	157	0
chr5	112838815	112838815	C	-	PASS	APC	Frame_Shift_Del		TO004	35	44	121	0
chr5	112839783	112839784	GA	-	PASS	APC	Frame_Shift_Del	novel	TO004	70	78	135	0
chr6	7585684	7585684	G	A	PASS	DSP	Missense_Mutation	rs755395389	TO004	204	220	534	0
chrX	154466502	154466502	C	T	PASS	PLXNA3	Nonsense_Mutation	novel	TO004	0	78	63	0
chr1	114713907	114713907	T	A	PASS	NRAS	Missense_Mutation	rs121913255	TO005	80	81	95	0
chr1	228288265	228288265	C	A	PASS	OBSCN	Missense_Mutation	rs760864720	TO005	131	123	193	0

chr13	10112465 6	10112465 6	C	T	PASS	NALCN	Missense_Mutation		TO005	173	10	88	0
chr2	18579213 1	18579213 1	G	T	PASS	FSIP2	Missense_Mutation	novel	TO005	137	123	112	0
chr5	32689147	32689147	C	T	PASS	NPR3	Missense_Mutation	rs14707922 41	TO005	78	87	83	0
chr5	32712185	32712185	G	A	PASS	NPR3	Missense_Mutation	rs56985044 8	TO005	351	19	211	0
chr5	11283852 4	11283852 4	G	T	PASS	APC	Missense_Mutation	novel	TO005	121	86	135	0
chr5	11283852 6	11283852 6	C	T	PASS	APC	Nonsense_Mutation		TO005	121	86	135	0
chr5	11284006 8	11284008 9	GCCACGGAAAGTACTCCAGATG	-	PASS	APC	Frame_Shift_Del	novel	TO005	78	71	109	0
chr7	21710684	21710684	C	T	PASS	DNAH11	Missense_Mutation	novel	TO005	98	71	73	0

chr8	3396299	3396299	C	T	PASS	CSMD1	Missense_Mutation	rs776627843	TO005	77	34	54	0
chrX	15446417 8	15446417 8	G	A	PASS	PLXNA3	Missense_Mutation	rs782421628	TO005	130	105	101	0
chr17	7673533	7673533	A	G	PASS	TP53	Splice_Site		TO008	0	37	52	0
chr2	17873099 3	17873099 3	C	A	PASS	TTN	Missense_Mutation	rs14191949 17	TO008	40	31	62	0
chr1	22827921 0	22827921 0	G	A	PASS	OBSCN	Missense_Mutation	rs11614070 94	TP002	106	32	115	0
chr1	23334649 1	23334649 1	G	T	PASS	MAP3K21	Missense_Mutation	novel	TP002	54	4	45	0
chr12	25245350	25245350	C	T	PASS	KRAS	Missense_Mutation	rs121913529	TP002	82	18	51	0
chr13	10129223 8	10129223 8	C	T	PASS	NALCN	Missense_Mutation		TP002	73	23	111	0

chr4	12531696 8	12531696 8	G	A	PASS	FAT4	Missense_Mutation		TP002	167	27	186	0
chr5	32712011	32712011	G	C	PASS	NPR3	Missense_Mutation	novel	TP002	98	17	193	0
chr5	11282891 9	11282891 9	C	T	PASS	APC	Nonsense_Mutation	rs137854574	TP002	30	8	46	0
chr6	7571983	7571983	G	A	PASS	DSP	Missense_Mutation		TP002	108	17	127	0
chr8	2973156	2973156	G	A	PASS	CSMD1	Missense_Mutation	rs13732956 87	TP002	97	9	82	0
chr1	23335386 8	23335386 8	C	G	PASS	MAP3K21	Missense_Mutation	novel	TP003	53	22	99	0
chr12	25245351	25245351	C	A	PASS	KRAS	Missense_Mutation	rs121913530	TP003	46	22	83	0
chr12	56087843	56087843	C	T	PASS	ERBB3	Missense_Mutation	rs14297845 31	TP003	54	23	93	0

chr13	101143106	101143106	C	T	PASS	NALCN	Missense_Mutation		TP003	276	31	139	0
chr13	101283953	101283953	G	A	PASS	NALCN	Missense_Mutation	rs549926959	TP003	28	77	51	0
chr17	7673610	7673610	T	C	PASS	TP53	Splice_Site	rs397516439	TP003	38	38	105	0
chr2	178584764	178584764	G	A	PASS	TTN	Missense_Mutation	novel	TP003	103	53	216	1
chr2	185801939	185801939	A	T	PASS	FSIP2	Missense_Mutation	novel	TP003	50	38	73	0
chr4	125415615	125415615	G	A	PASS	FAT4	Missense_Mutation		TP003	117	87	216	0
chr4	152326137	152326137	G	C	PASS	FBXW7	Missense_Mutation	rs149680468	TP003	78	34	142	0
chr5	112839515	112839519	AAAAG	-	PASS	APC	Frame_Shift_Del	rs121913224	TP003	13	93	97	0
chr7	21591523	21591523	A	-	PASS	DNAH11	Frame_Shift_Del	novel	TP003	59	26	64	0

chr12	25245348	25245348	C	A	PASS	KRAS	Missense_Mutation	rs121913535	TP004	78	8	58	0
chr17	7674220	7674220	C	T	PASS	TP53	Missense_Mutation	rs11540652	TP004	89	10	136	0
chr18	51065549	51065549	G	A	PASS	SMAD4	Missense_Mutation	rs377767347	TP004	94	16	160	0
chr4	152323074	152323074	C	T	PASS	FBXW7	Missense_Mutation		TP004	124	22	157	0
chr4	152328232	152328232	C	T	PASS	FBXW7	Missense_Mutation	rs1057519895	TP004	120	14	157	0
chr5	112838815	112838815	C	-	PASS	APC	Frame_Shift_Del		TP004	123	11	103	0
chr5	112839783	112839784	GA	-	PASS	APC	Frame_Shift_Del	novel	TP004	134	25	150	0
chr6	7585684	7585684	G	A	PASS	DSP	Missense_Mutation	rs755395389	TP004	412	73	523	0

chrX	154466502	154466502	C	T	PASS	PLXNA3	Nonsense_Mutation	novel	TP004	21	30	44	0
chr1	114713907	114713907	T	A	PASS	NRAS	Missense_Mutation	rs121913255	TP005	72	18	99	0
chr1	228288265	228288265	C	A	PASS	OBSCN	Missense_Mutation	rs760864720	TP005	155	30	180	0
chr17	7674947	7674948	-	T	PASS	TP53	Frame_Shift_Ins	novel	TP005	124	8	165	0
chr2	185792131	185792131	G	T	PASS	FSIP2	Missense_Mutation	novel	TP005	136	16	178	0
chr4	125317219	125317219	C	A	PASS	FAT4	Missense_Mutation	novel	TP005	180	5	185	0
chr5	32689147	32689147	C	T	PASS	NPR3	Missense_Mutation	rs1470792241	TP005	64	20	92	0
chr5	112838524	112838524	G	T	PASS	APC	Missense_Mutation	novel	TP005	115	26	101	0

chr5	112838526	112838526	C	T	PASS	APC	Nonsense_Mutation		TP005	115	26	101	0
chr5	112840068	112840089	GCCACGGAAAGTACTCCAGATG	-	PASS	APC	Frame_Shift_Del	novel	TP005	93	13	139	0
chr7	21710684	21710684	C	T	PASS	DNAH11	Missense_Mutation	novel	TP005	94	18	127	0
chr8	3396299	3396299	C	T	PASS	CSMD1	Missense_Mutation	rs776627843	TP005	64	6	82	0
chrX	154464178	154464178	G	A	PASS	PLXNA3	Missense_Mutation	rs782421628	TP005	106	24	141	0

Supplementary Figures

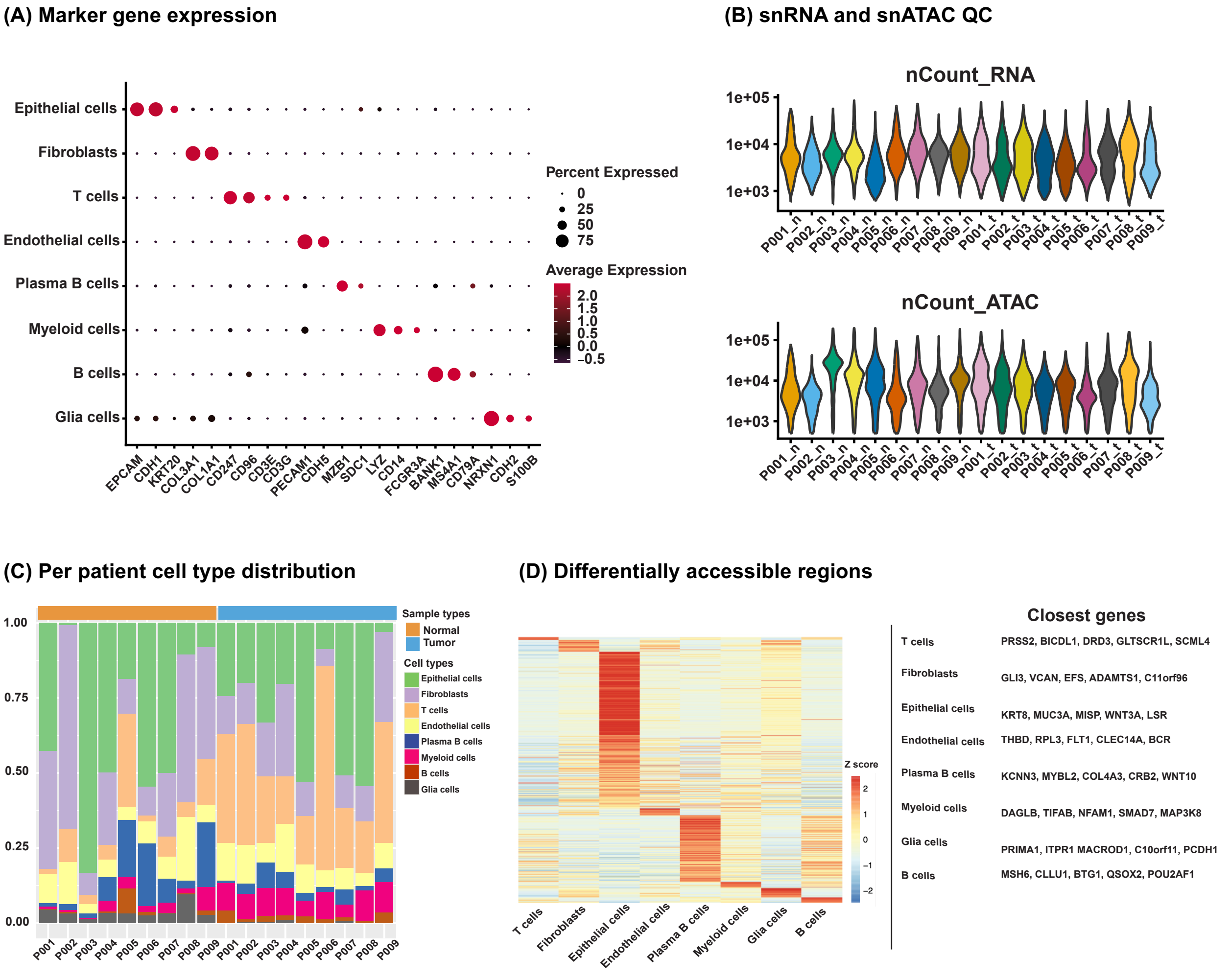

Figure S1 Identification and quantification of major cell types in colorectal tissues.

(A). Dot plot showing selected marker genes for major cell types. Dot size reflects the percentage of cells expressing each marker, and the color represents average expression relative to all cell types. **(B)**. Violin plot displays quality control metrics: UMI counts for snRNA-seq data (top panel) and total number of fragments in peaks (Tn5 insertion frequency) for snATAC-seq data (bottom panel). QC, quality control. **(C)**. Bar plot displaying the relative abundance of each cell type across patients. The x-axis represents each patient, with normal (orange) and tumor (blue) samples. The y-axis represents percentage per cell type. **(D)**. Heatmap depicting average Tn5 insertion frequency across differentially accessible regions (DARs) for each cell type (left panel). Rows are scaled z-score of insertion frequency. The right panel lists the top closest genes associated with each cluster.

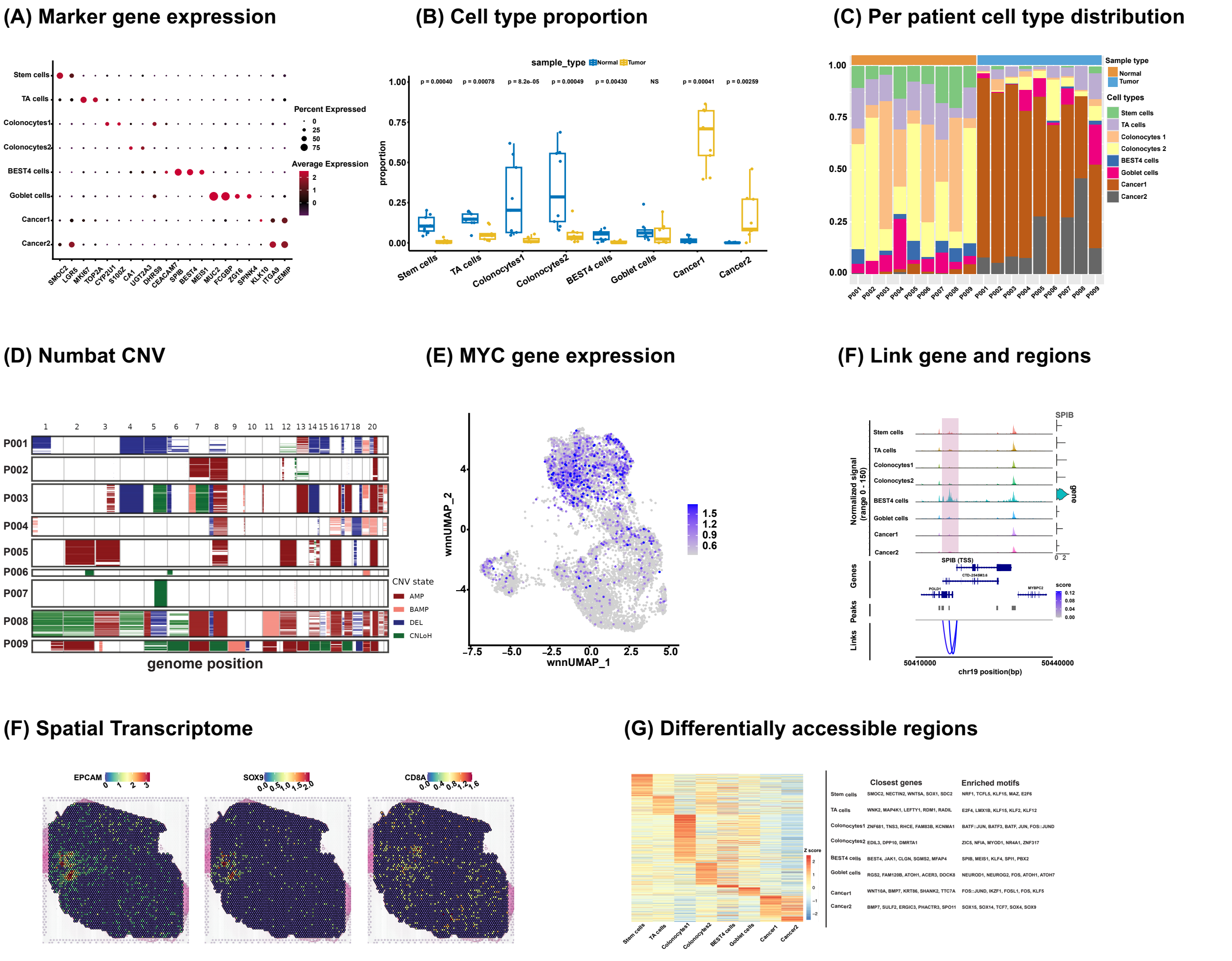

Figure S2 Colorectal tumor cells.

(A). Dot plot showing selected marker genes across epithelial subclusters. Dot size indicates the percentage of cells expressing each marker, and color represents average expression relative to all subclusters. **(B)**. Box plot showing the proportion of epithelial subtypes in normal (blue) and tumor (yellow) samples across patients, each dot represents a patient. Statistical significance was assessed using the Wilcoxon test (NS, not significant). **(C)**. Bar plot displaying the proportion of annotated epithelial subclusters across patients. The x-axis represents each patient, colored by sample type (orange: normal and blue: tumor). The y-axis represents the relative percentages. **(D)**. Inferred copy number variation (iCNV) landscape and reconstructed phylogeny across CRC patients. Each rectangle row represents a patient and columns to chromosomes. Copy number events include amplification (AMP), balanced amplification (BAMP), deletion (DEL) and copy-neutral loss of heterozygosity (CNLoH). **(E)**. UMAP projection of *MYC* expression across epithelial cells. Color scale represents normalized log-fold changes. **(F)**. Spatial transcriptome showing normalized gene expression of *EPCAM*, *SOX9* and *CD8A*, highlighting regions with elevated expressions. **(G)**. Heatmap depicts the average Tn5 insertion frequency across differentially accessible regions (DARs) for each epithelial subcluster (left panel). Rows are scaled z-score of insertion frequency. Significant closest genes and enriched motifs for each subcluster are listed in the right panel. **(H)**. Chromatin accessibility peaks linked with *SPIB* gene expression across ± 20 kb of gene body. High-confidence regulatory regions (adjusted for biases with peak size, GC content and overall accessibility) are highlighted in pink. The transcription start site (TSS) is indicated.

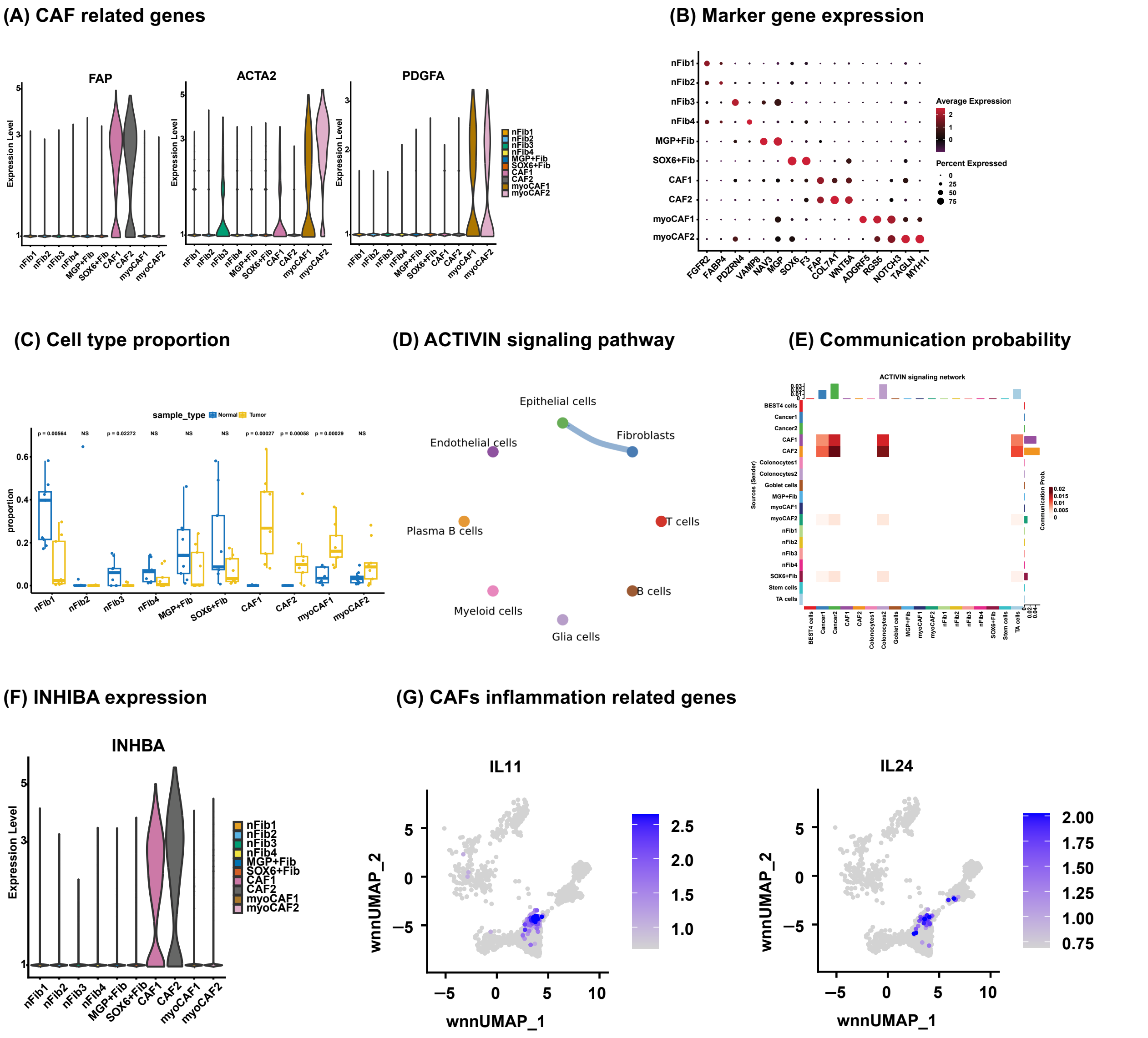

Figure S3 Colorectal fibroblasts.

(A). Violin plots depict the expression levels of *FAP*, *ACTA2* and *PDGFA* genes within the fibroblast compartment. **(B)**. Dot plot presents the selected marker genes across fibroblast subclusters. Dot size indicates the percentage of cells expressing each marker, color represents average expression relative to all subclusters. **(C)**. Box plots quantifying the proportion of fibroblast subclusters in normal (blue) and tumor (yellow)samples, each dot represents a patient. Statistical significance was calculated using the Wilcoxon test (NS, not significant). **(D)**. Circle plot displaying inferred activin signaling interactions among cell types in tumor samples. Nodes represent cell types, edge width represents the number of potential ligand-receptor pairs, and edge color corresponds to the ligand-sending cell type. **(E)**. Heatmap displaying inferred activin signaling network between epithelial and fibroblast subclusters in tumor samples. The X-axis displays potential receptor receivers, and the y-axis displays potential ligand senders. Color scale represents communication probability. **(F)**. Violin plot depicts expression of *INHBA* gene across fibroblast subclusters. **(G)**. UMAP projection of *IL11* and *IL24* expression in tumor fibroblasts. Color scale represents normalized log-fold changes.

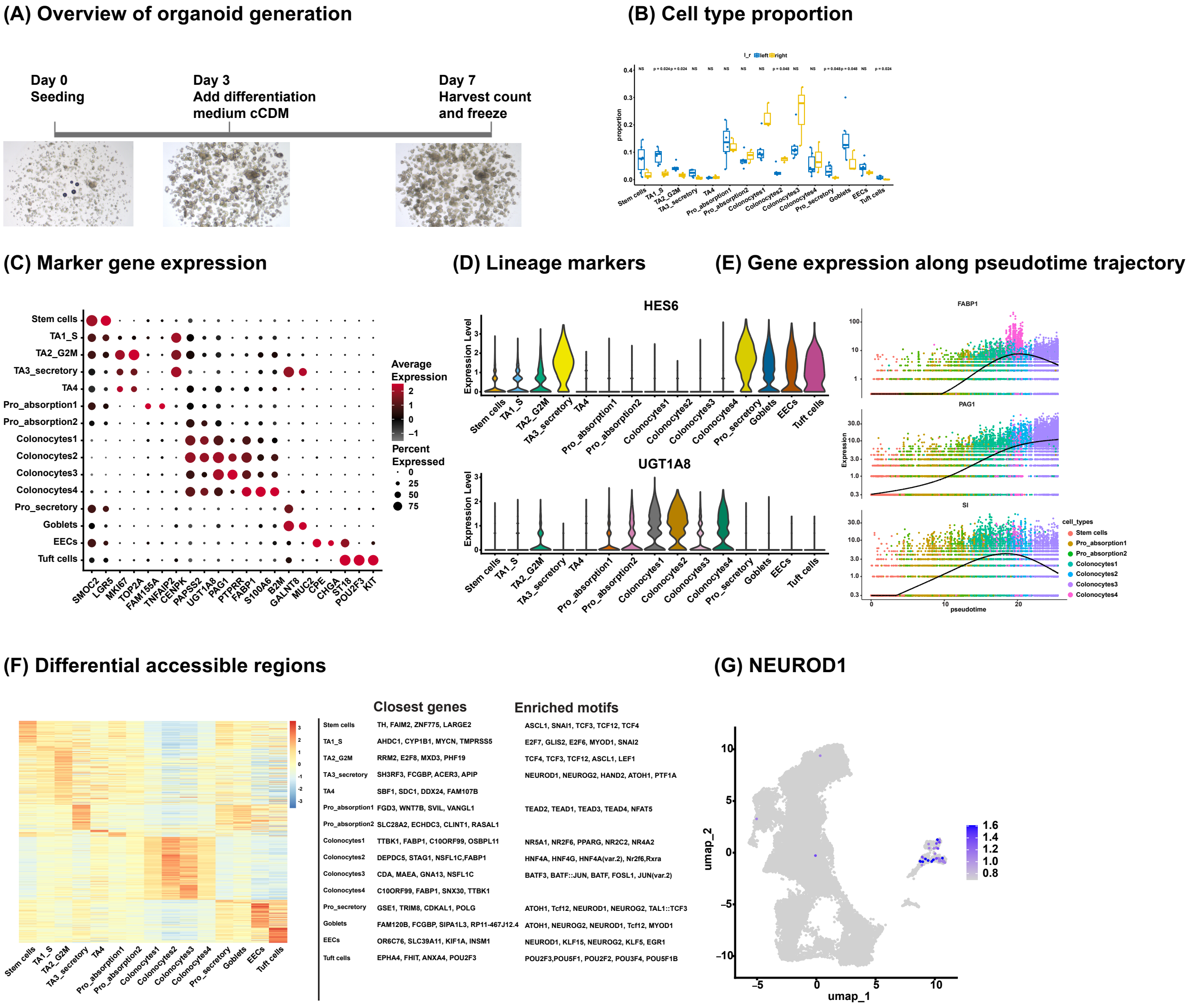

Figure S4 Colorectal N-PDOs.

(A). Schematic overview of PDO culture. PDOs were initiated in amplification medium (colon normal medium, CNM or colon tumor medium, CTM) on Day 0, changed to differentiation medium (combination colon differentiation medium, cCDM) on Day 3 and harvested on Day 7. **(B)**. Box plots quantifying the proportion of colorectal N- PDOs in left-sided (blue) and right-sided (yellow) colorectal samples, each dot represents a patient. Statistical significance was calculated using the Wilcoxon test (NS, not significant). **(C)**. Dot plot displays selected marker genes across colorectal N-PDOs subclusters. Dot size represents the percentage of cells expressing each marker, color represents average expression relative to all subclusters. **(D)**. Violin plots display the expression levels of *HES6* and *UGT1A8* genes across subclusters of colorectal N-PDOs. **(E)**. Gene expression dynamics of *FABP1*, *PAG1* and *SI* across the pseudo-time trajectory for absorptive lineage-related clusters (including stem cells, pro_absorption1-2 and colonocytes1-4). Cells are colored by cell types and the black lines indicate average gene expression trends. **(F)**. Heatmap depicts the average Tn5 insertion frequency across differentially accessible regions (DARs) for each N-PDO subclusters (left panel). Rows are scaled z-score of insertion frequency. Significant closest genes and enriched motifs for each subcluster are listed in the right panel (if applicable). **(G)**. UMAP projection of *NEUROD1* expression across N-PDOs. Color scale represents normalized log-fold changes.

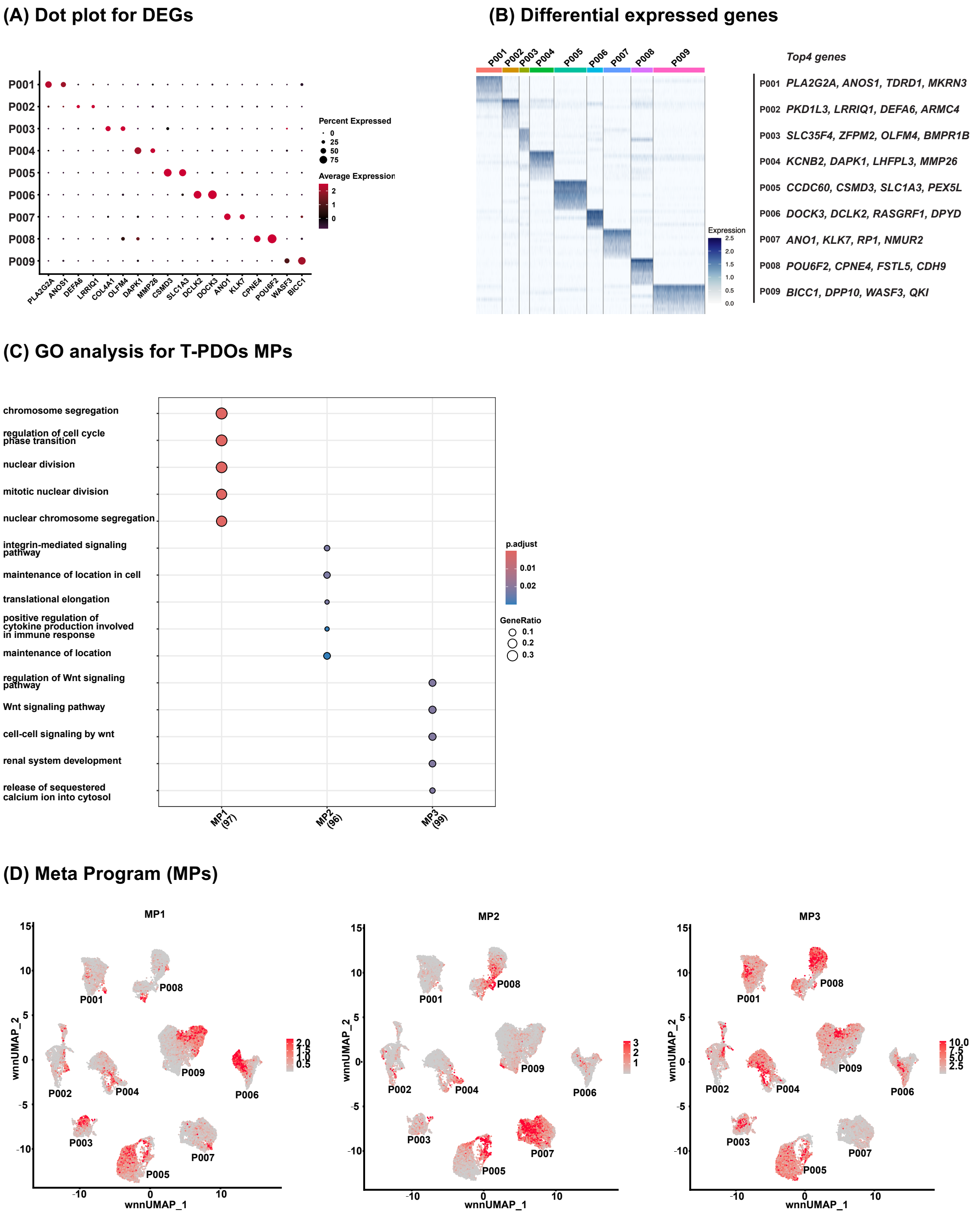

Figure S5 Colorectal T-PDOs.

(A). Dot plot quantifying selected marker genes across patients. Dot size indicates the percentage of cells expressing each marker, and color represents the average expression relative to all patients. **(B)**. Heatmap displaying differential expression genes (DEGs) across patients, with columns representing individual patient cells and rows corresponding to the top 10 genes. The four most highly expressed genes are highlighted in the right panel. **(C)**. Gene ontology (GO) analysis showing GOs specific for MP1-MP3 meta-programs. Color scale represents the adjusted p-value (Benjamini FDR < 0.05), and circle size represents the gene ratio. **(D)**. UMAP projection of MP1-MP3 gene signatures in T-PDOs. Color scale represents normalized log-fold changes in signature expression.

(A) Predicted Cell Type per T-PDO

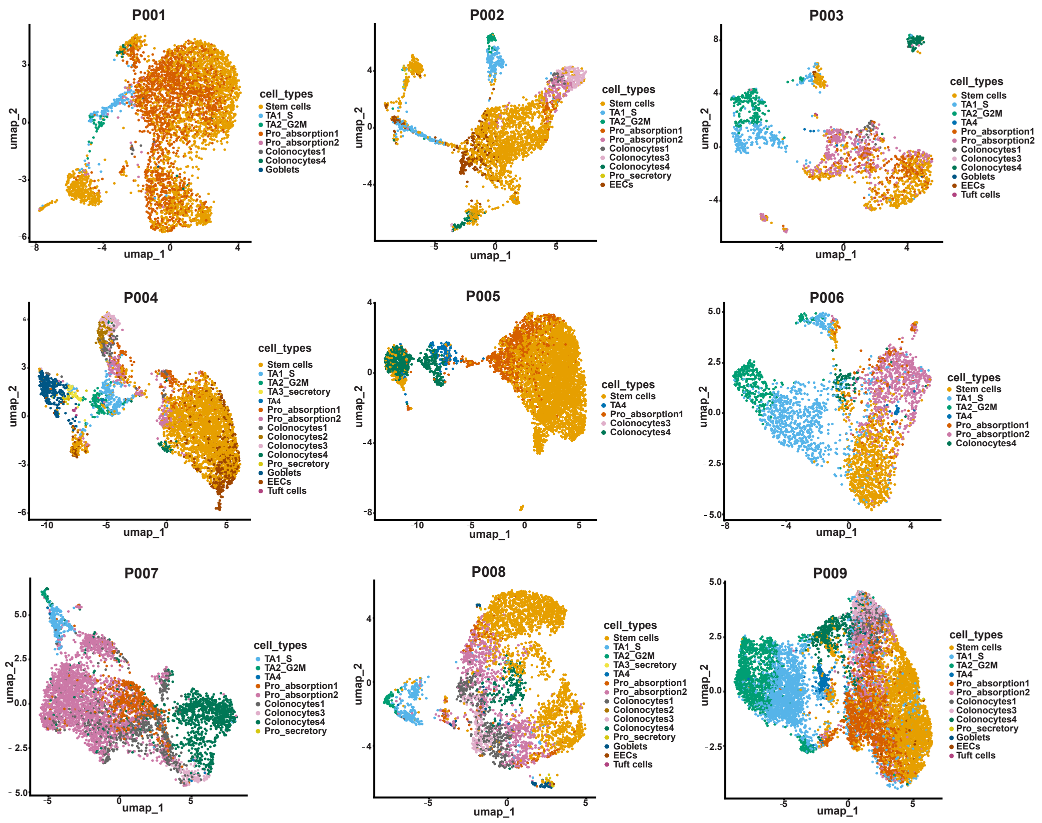

(B) Predicted Cell Type Composition Across T-PDOs

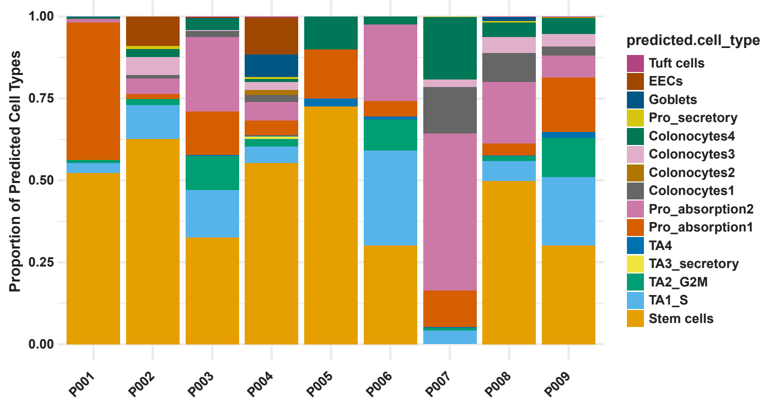

Figure S6 Predicted cell-types of colorectal T-PDOs.

(A). UMAP projection of T-PDO snRNA-seq data across patients, colored by predicted cell-types. Abbreviations including: TA1_S (transit amplifying cells in S phase), TA2_G2M (transit amplifying cells in G2M phase), TA3_secretory (secretory lineage related transit amplifying cells), Pro (progenitor state), EECs (enteroendocrine cells). Cells are colored by predicted cell-types (if applicable). **(B).** Bar plot illustrating the proportion of predicted cell-types across patients. The x-axis represents each patient, and the y-axis represents the relative percentages.

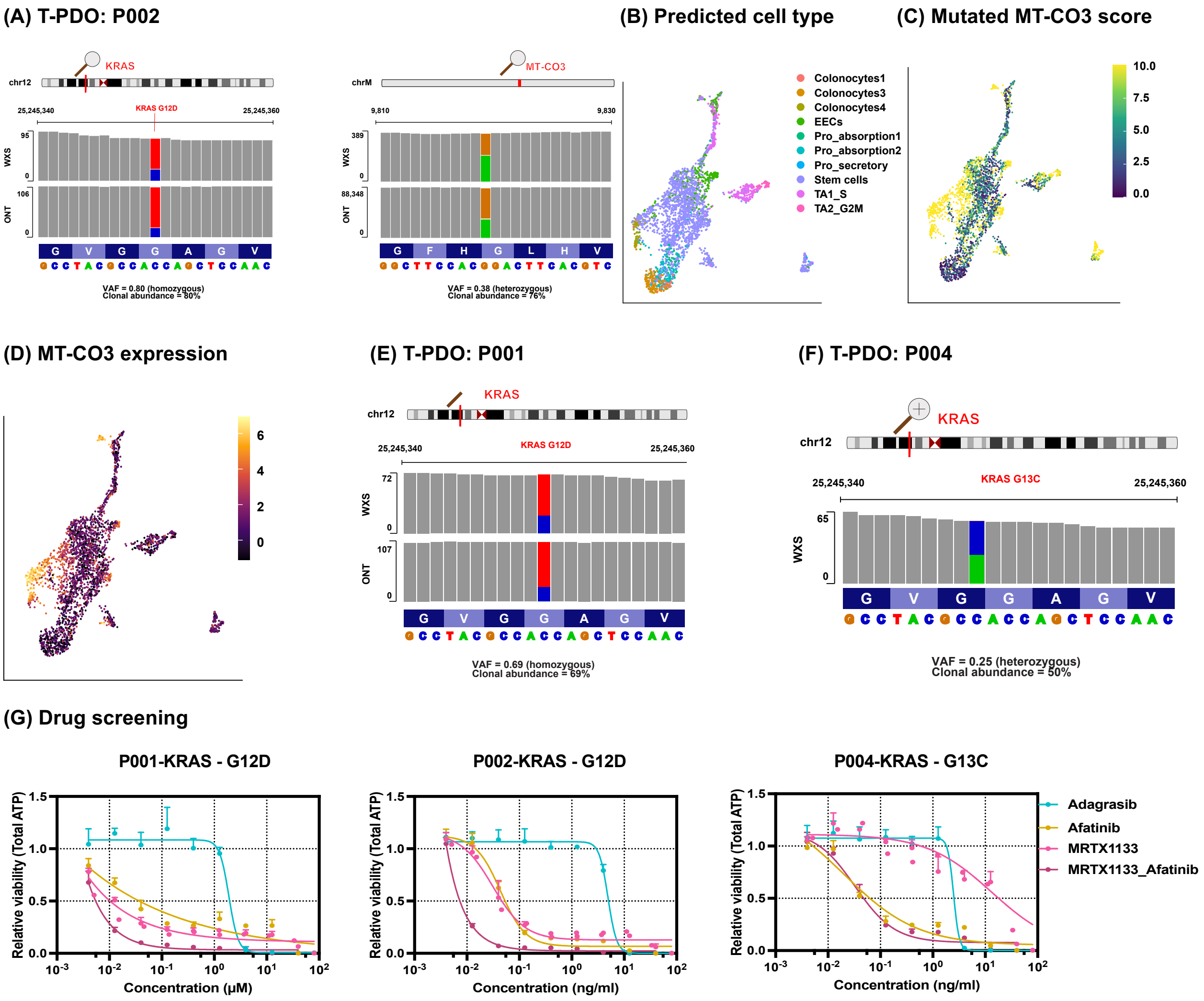

Figure S7 Somatic mutation and drug screening of colorectal T-PDOs

(A). Left panel: P002 *KRAS*-G12D mutation on chromosome 12 shown with WXS and ONT read alignments. VAF=0.80, indicating a homozygous event with 80% clonal abundance. Right panel: a presumed heterozygous mutation in *MT-CO3* (chrM) shown with WXS and ONT read alignments. VAF-0.38, and clonal abundance =76%. **(B)-(D)**. UMAP projection of T-PDO of P002, colored with predicted cell-types **(B)**, mutated MT-CO3 score **(C)** and MT-CO3 expression **(D)**. **(E)**. P001 *KRAS*-G12D mutation on chromosome 12 shown with WXS and ONT read alignments. VAF=0.69, suggesting a homozygous event with 69% clonal abundance. **(F)**. P004 *KRAS*-G13C mutation on chromosome 12 shown with WXS read alignments. VAF=0.25, suggesting a heterozygous event with 50% clonal abundance. **(G)**. Dose-response curves show relative viability (total ATP) of T-PDOs for P001 (*KRAS*-G12D), P002 (*KRAS*-G12D) and P004 (*KRAS*-G13C) treated with Adagrasib, MRTX1133, Afatinib and their combinations. PDOs were exposed to increasing concentrations of single or combination treatments. Error bars represent the standard deviation of technical replicate.

Supplementary references

1 Sato, T. *et al.* Long-term expansion of epithelial organoids from human colon, adenoma, adenocarcinoma, and Barrett's epithelium. *Gastroenterology* **141**, 1762-1772 (2011). https://doi.org/10.1053/j.gastro.2011.07.050

2 Huang, X. & Huang, Y. Cellsnp-lite: an efficient tool for genotyping single cells. *Bioinformatics* **37**, 4569-4571 (2021). https://doi.org/10.1093/bioinformatics/btab358

3 Huang, Y., McCarthy, D. J. & Stegle, O. Vireo: Bayesian demultiplexing of pooled single-cell RNA-seq data without genotype reference. *Genome Biol* **20**, 273 (2019). https://doi.org/10.1186/s13059-019-1865-2

4 Fleming, S. J. *et al.* Unsupervised removal of systematic background noise from droplet-based single-cell experiments using CellBender. *Nat Methods* **20**, 1323-1335 (2023). https://doi.org/10.1038/s41592-023-01943-7

5 McGinnis, C. S., Murrow, L. M. & Gartner, Z. J. DoubletFinder: Doublet Detection in Single-Cell RNA Sequencing Data Using Artificial Nearest Neighbors. *Cell Syst* **8**, 329-337 e324 (2019). https://doi.org/10.1016/j.cels.2019.03.003

6 Hao, Y. *et al.* Dictionary learning for integrative, multimodal and scalable single-cell analysis. *Nat Biotechnol* **42**, 293-304 (2024). https://doi.org/10.1038/s41587-023-01767-y

7 Wu, T. *et al.* clusterProfiler 4.0: A universal enrichment tool for interpreting omics data. *Innovation (Camb)* **2**, 100141 (2021). https://doi.org/10.1016/j.xinn.2021.100141

8 Liberzon, A. *et al.* The Molecular Signatures Database (MSigDB) hallmark gene set collection. *Cell Syst* **1**, 417-425 (2015). https://doi.org/10.1016/j.cels.2015.12.004

9 Wu, Y., Tamayo, P. & Zhang, K. Visualizing and Interpreting Single-Cell Gene Expression Datasets with Similarity Weighted Nonnegative Embedding. *Cell Syst* **7**, 656-666 e654 (2018). https://doi.org/10.1016/j.cels.2018.10.015

10 Cao, J. *et al.* The single-cell transcriptional landscape of mammalian organogenesis. *Nature* **566**, 496-502 (2019). https://doi.org/10.1038/s41586-019-0969-x

11 Jin, S. *et al.* Inference and analysis of cell-cell communication using CellChat. *Nat Commun* **12**, 1088 (2021). https://doi.org/10.1038/s41467-021-21246-9

12 Gao, T. *et al.* Haplotype-aware analysis of somatic copy number variations from single-cell transcriptomes. *Nat Biotechnol* **41**, 417-426 (2023). https://doi.org/10.1038/s41587-022-01468-y

13 Hao, Y. *et al.* Integrated analysis of multimodal single-cell data. *Cell* **184**, 3573-3587 e3529 (2021). https://doi.org/10.1016/j.cell.2021.04.048

14 Jia, P. *et al.* MSIsensor-RNA: Microsatellite Instability Detection for Bulk and Single-cell Gene Expression Data. *Genomics, Proteomics & Bioinformatics* (2024). https://doi.org/10.1093/gpbjnl/qzae004

15 Stuart, T., Srivastava, A., Madad, S., Lareau, C. A. & Satija, R. Single-cell chromatin state analysis with Signac. *Nat Methods* **18**, 1333-1341 (2021). https://doi.org/10.1038/s41592-021-01282-5

16 Zhang, Y. *et al.* Model-based analysis of ChIP-Seq (MACS). *Genome Biol* **9**, R137 (2008). https://doi.org/10.1186/gb-2008-9-9-r137

17 Muto, Y. *et al.* Single cell transcriptional and chromatin accessibility profiling redefine cellular heterogeneity in the adult human kidney. *Nat Commun* **12**, 2190 (2021). https://doi.org/10.1038/s41467-021-22368-w
